# Infrared markers of topological phase transitions in quantum spin Hall insulators

**DOI:** 10.1038/s41524-025-01780-6

**Published:** 2025-10-16

**Authors:** Paolo Fachin, Francesco Macheda, Paolo Barone, Francesco Mauri

**Affiliations:** 1 Dipartimento di Fisica, Sapienza Università di Roma, Roma, Italy; 2 CNR-SPIN, Area della Ricerca di Tor Vergata, Roma, Italy

**Keywords:** Topological insulators, Electronic properties and materials, Topological matter, Two-dimensional materials

## Abstract

Using first principles techniques, we show that infrared optical response allows us to discriminate between the topological and the trivial phases of 2D quantum spin Hall insulators (QSHI). We showcase germanene and jacutingaite, of recent experimental realization, as prototypical systems where the infrared spectrum is discontinuous across the transition, due to sudden and large discretized jumps of the Born effective charges (up to ~2). Our results, rationalized thanks to the low-energy Kane–Mele model, are robust with respect to dynamical effects, relevant when the electronic energy gap is comparable with the phonon frequency. In the small gap QSHI germanene, due to dynamical effects, the in-plane phonon resonance in the optical conductivity shows a Fano profile with remarkable differences in the intensity and the shape between different phases. Instead, the large-gap QSHI jacutingaite presents several IR-active phonon modes whose spectral intensities drastically change between different phases.

## Introduction

Topological quantum spin Hall insulators (QSHIs), firstly introduced by Kane and Mele^1^, present an even number of helical edge states protected by time-reversal symmetry^2–6^. These counterpropagating modes of opposite helicity realize one-dimensional wires where the suppression of backscattering due to spin-momentum locking and time-reversal symmetry allows for dissipationless currents, with the possibility of promising technological devices such as topological field-effect transistors^7–9^. Even though the Kane–Mele model was ideated for graphene, in practice, this is not a QSHI because the spin-orbit coupling inducing non-trivial band inversion is too weak. Nonetheless, graphene-like monoelemental 2D honeycomb as germanene with a buckled structure^10^ still realizes the Kane–Mele model, ensuring the possibility to tune topological phases thanks to the application of an orthogonal electric field^11^. In addition, the germanene monolayer has been shown to host a QSHI state with a sufficiently large gap such that topological properties are robust even at room temperature^10,12,13^, which is essential for technological applications. An electric-field tunable topological state, so far detected in germanene^10^ and Na_3_Bi^14^ in a configuration where the scanning tunneling microscope (STM) allows to apply a gate field, finds promising application in the realization of the topological field-effect transistor^9,15^. In this device, the “ON” and “OFF” states correspond to the presence or the absence of topologically protected ballistic transport along the edge channels of the 2D insulators.

The quest for a large gap, stable at ambient conditions, and electric-field tunable QSHI, allowing for room temperature dissipationless electronic transport, found a promising candidate in jacutingaite (Pt_2_HgSe_3_), a naturally occurring layered mineral, first discovered in 2008^16^ and later also artificially synthesized^17^. In its bulk phase, jacutingaite displays a peculiar dual topology where a topological crystalline phase is coexisting with a weak ${{\mathcal{Z}}}_{2}$ topological state^18–20^. The exfoliated monolayer is predicted to be a large-gap Kane–Mele QSHI whose topological properties are described by a buckled Kane–Mele model, analogous to the one of germanene, realized by the Hg s orbitals^21^. Exfoliation to a few layers^22^ suggests the possibility of realizing a large-gap QSHI monolayer system, which can be even integrated in heterostructures preserving its topological properties^21^.

The detection of QSHI states has been based so far on either charge transport measurements directly probing the longitudinal resistance in Hall bar geometry^23–29^ or discriminating conductive edge states from insulating bulk by directly imaging the local conductivity with STM measures^10,12–14,22,30–43^. Supporting evidence for the presence of topological phases has also been proposed by comparing ab initio band structures with single-particle spectral functions measured via angle-resolved photoemission spectroscopy (ARPES)^31,32^. Despite not providing a direct characterization of topological properties, this technique has been used to show the topological gap reduction due to the electric field in Na_3_Bi^14^. Analogously, the presence of a finite local density of states (LDOS) on the edge states of a system probed by the STM measures sometimes does not provide a sufficient evidence for the existence of a topological non-trivial state as compared to the measure of ballistic edge conductivity, which in instead a robust signature of QSHI phase, but more articulate to measure. As an illustrative example, nanoscale four-tip STM transport measurements performed on (Bi_0.16_Sb_0.84_)_2_Te_3_ showed that the observation of an edge state in the LDOS does not guarantee the presence of ballistic conductance in the edge states, the necessary condition for a QSHI state^44^. Thus, since both STM and ARPES cannot provide an indisputable proof of the presence of topological states, the experimental identification of QSHI states is still a challenge, motivating the seek for either improvements in the already used techniques or for alternative methods^9^. Some theoretical proposals suggest plasmon-based detections^45,46^ or exploiting the Ruderman-Kittel-Kasuya-Yosida interaction with magnetic impurities^47^, as well as on the discontinuous changes of piezoelectric response in 2D time-reversal invariant systems^48,49^ thanks to its relation with the valley Chern number or to the QSHI phase topological index ${{\mathcal{Z}}}_{2}$. A similar connection also holds for the Born effective charges in the prototypical Haldane and Kane–Mele models^50^, exhibiting a discrete jump between nearly vanishing values in the topological phases and large finite ones in the trivial states. Since the Born effective charges quantify the intensity of the vibrational contribution in infrared (IR) spectra, optical spectroscopy could provide a detection method for topological phase transitions.

In this work, we use ab initio calculations to support the feasibility of the proposal by evaluating the optical response of two real QSHI materials, germanene and jacutingaite. We here study the topological phase transition by tuning an external electric field, originating from a field-effect setup configuration. We first address germanene, a direct realization of the Kane–Mele model, and then move to the jacutingaite monolayer, where the large number of atoms enriches the picture of the Kane–Mele model, which still faithfully accounts for its low-energy physical properties. Finally, we test the robustness of our findings by including dynamical effects that come into play whenever phonon frequencies are in resonance with the energy gap of the material^51,52^. Since the topological transition must occur across a metallic point, this resonance regime will always appear near the phase transition where the closing and reopening of the gap occurs. While dynamical effects are found to smoothen the discontinuous jump of the Born effective charges, vibrational contribution to the infrared spectra still presents distinct features providing a direct marker for the topological phase transition.

## Results

### Theoretical framework

#### Infrared spectroscopy

The optical conductivity is used to assess the response of the system to electromagnetic fields. In general, the conductivity is determined by two distinct contributions as

1 $${\sigma }_{\alpha \beta }(\omega )={\sigma }_{\alpha \beta }^{{\rm{el}}}(\omega )+{\sigma }_{\alpha \beta }^{{\rm{ion}}}(\omega ),$$

where ${\sigma }_{\alpha \beta }^{{\rm{el}}}(\omega )$ accounts for electronic excitations at clamped nuclei, while ${\sigma }_{\alpha \beta }^{{\rm{ion}}}(\omega )$ has resonances at the vibrational excitations of the system^51^. We here concentrate on the expression of the ionic contribution, which reads

2 $${\sigma }_{\alpha \beta }^{\,\text{ion}\,}(\omega )=\frac{-i\omega }{A}\sum _{\nu }\frac{{f}_{\nu ,\alpha }(\omega ){f}_{\nu ,\beta }(\omega )}{{\omega }_{\nu }^{2}-{(\omega +i{\gamma }_{\nu }/2)}^{2}}.$$

where *A* is the area of the unit cell of the two-dimensional material, *ω*_*ν*_ are the phonon frequencies with full width at half maximum *γ*_*ν*_ in the quasiparticle approximation. The oscillator strengths that determine the intensity of the infrared absorption are expressed as

3 $${f}_{\nu ,\alpha }(\omega )=e\sum _{s,\delta }{Z}_{s,\alpha \delta }^{* }(\omega )\frac{{e}_{\nu ,\delta }^{s}}{\sqrt{{M}_{s}}}$$

where *M*_*s*_ is the ionic mass, ${e}_{\nu ,\delta }^{s}$ is the phonon polarization vector, and ${Z}_{s,\alpha \delta }^{* }(\omega )$ are the Born effective charges, whose expression in the framework of time-dependent density functional theory^52^ is reported in the SI (see Supplementary Material at URL will be inserted by publisher for additional details, which include refs. ^1,11,50,51,53–56^). The Born effective charges are the sum of a term accounting for the rigid displacement of the ionic and electronic charge (Z^*,rig^) and an “anomalous” term describing the polarization of the electronic charge density induced by the lattice vibrations (Z^*,an^), i.e., Z^*^ = Z^*,rig^ + Z^*,an^. In other words, the first term represents the bare ionic (pseudo)charge, while the second comes from the electronic polarization induced by an atomic displacement in the adiabatic approximation.

We now specialize in two-dimensional systems. For in-plane components, it generally holds ∣Z^*,rig^∣ ≪ ∣Z^*,an^∣^50^, while generally out-of-plane components respect ∣Z^*,an^∣ ≪ ∣Z^*,rig^∣^51^. In the following, we will mostly concentrate our attention on the in-plane components of Born effective charges and discuss their behavior in terms of the anomalous component. In the static limit (*ω* = 0), this is expressed in terms of the Berry curvature as^50^

4 $${Z}_{s,\beta \alpha }^{* ,{\rm{an}}}=\frac{A}{{(2\pi )}^{2}}{\int}_{{\rm{BZ}}}{d}^{2}{\bf{k}}{\Omega }_{{k}_{\alpha }{u}_{s\beta }}({\bf{k}}).$$

In sections “Germanene” and “ Jacutingaite,” we will study the infrared response of germanene and jacutingaite in the static limit through ab initio simulation and compare to predictions performed with the Kane-Male model. Here, the approximation is that the energy of the vibrations is smaller than the typical energy gap of the material, or in other words, that ${\sigma }_{\alpha \beta }^{{\rm{el}}}({\omega }_{\nu }) \sim 0$ and that ${Z}_{s,\alpha \delta }^{* }({\omega }_{\nu }) \sim {Z}_{s,\alpha \delta }^{* }(\omega =0)$. On the contrary, in the section “Dynamical effects,” we will explicitly lift the static approximation and quantify the impact of the electronic resonances on the infrared features of the vibrational modes within the Kane–Mele model.

#### Kane–Mele model

The inclusion of spin-orbit coupling in a next-nearest-neighbor tight-binding model description of a 2D monoelemental honeycomb lattice naturally gives rise to topologically non-trivial quantum spin hall states described by the topological index ${{\mathcal{Z}}}_{2}$, equal to ±1 in topological phases and 0 in the trivial ones^1,2^. Originally introduced for graphene, where the weak spin-orbit coupling prevents the realization of the topological non-trivial state, the model is straightforwardly extended to a lattice with a buckled structure, such as germanene and other monoelemental xenes (silicene, stanene^53^). With the aid of Wannier functions techniques, the model has been shown to also describe the relevant low-energy physics of jacutingaite^21^.

In our model Hamiltonian, we introduce an effective electric field ${E}_{z}^{{\rm{eff}}}$ along the out-of-plane direction^10,11,21,57^, which breaks inversion symmetry between the sublattices. In fact, since the structure is buckled, ${E}_{z}^{{\rm{eff}}}$ introduces an energy difference $2ed{E}_{z}^{{\rm{eff}}}$ between the two sublattices, where *d* is the half buckling height and *e* the electronic charge, inducing an effective on-site energy that allows the system to perform transitions between the topological and the trivial phases. Our effective model Hamiltonian finally reads

5 $$\begin{array}{lll}{H}_{{\rm{el}}} &=& ed{E}_{z}^{{\rm{eff}}}\sum _{i\rho }{l}_{i}{c}_{i,\rho }^{\dagger }{c}_{i,\rho }-{t}_{1}\sum _{\rho }\sum _{\langle ij\rangle }{c}_{i,\rho }^{\dagger }{c}_{j,\rho }\\&&+\,i\frac{{\lambda }_{{\rm{SO}}}}{3\sqrt{3}}\sum _{\rho {\rho }^{{\prime} }}\sum _{\langle \langle ij\rangle \rangle }{l}_{ij}{c}_{i,\rho }^{\dagger }{({\sigma }_{z}^{{\rm{S}}})}_{\rho {\rho }^{{\prime} }}{c}_{j,{\rho }^{{\prime} }}.\end{array}$$

${c}_{i\rho },{c}_{i\rho }^{\dagger }$ are the electronic creation and annihilation operators, where *i* is a short-hand notation indicating both the cell and the sublattice index, *ρ* = {*↑*, *↓*} is the spin index, and *l*_*i*_ = {1, −1} is the sublattice index of the site *i*. 〈〉 means sum on first nearest neighbors and 〈〈〉〉 on the second ones. *d* is the half buckling height, *t*_1_ the nearest-neighbor hopping, *λ*_SO_ the diagonal spin-orbit coupling, ***σ***^*S*^ is the vector containing Pauli matrices in the spin space, *l*_*ij*_ = ±1 depending on the direction of the vector connecting the sites^2,58^. The system presents two direct energy gaps at ***K*** and ${{\boldsymbol{K}}}^{{\prime} }$.

The trigonal symmetry of the buckled system enforced by the electric field allows for additional Rashba terms^59^ that are however, negligible for the effects we will discuss. In detail, the absence of in-plane mirror symmetry allows for a further intrinsic Rashba term^11,53^, which is, though, negligible since it vanishes at the ***K*** and ${{\boldsymbol{K}}}^{{\prime} }$ points and the coupling constant is weak (see Supplementary Material at URL will be inserted by publisher for additional details, which include refs. ^1,11,50,51,53–56^). The extrinsic Rashba term usually connected with external electric fields or coupling with the substrate, is also much smaller than the spin-orbit coupling, leaving the unperturbed system in the topological phase and affecting the electric-field-induced topological transition in a negligible way, as discussed in refs. ^1,53^.

The low-energy approximation of the Kane–Mele model, obtained by expanding the Hamiltonian for small quasi-momenta ***p*** around the ***K*** and ${{\boldsymbol{K}}}^{{\prime} }$ points, leads to^11^

6 $${H}_{\eta }=\hslash {v}_{{\rm{F}}}({p}_{x}{\sigma }_{x}^{{\rm{P}}}-\eta {p}_{y}{\sigma }_{y}^{{\rm{P}}})+(ed{E}_{z}^{{\rm{eff}}}-\eta {\lambda }_{{\rm{SO}}}{\sigma }_{z}^{{\rm{S}}}){\sigma }_{z}^{{\rm{P}}},$$

where $\hslash {v}_{{\rm{F}}}=\frac{\sqrt{3}{t}_{1}a}{2}$ is the Fermi velocity and *a* is the lattice parameter, ${\sigma }_{z}^{{\rm{P}}}$ are the Pauli matrices in the pseudospin space describing the sublattice degree of freedom, and *η* = ±1 is the valley index (***K*** or ${{\boldsymbol{K}}}^{{\prime} }$ respectively). The energy spectrum around the ***K*** and ${{\boldsymbol{K}}}^{{\prime} }$ points is ${E}_{\eta }({\boldsymbol{p}})=\pm \sqrt{{\hslash }^{2}{v}_{{\rm{F}}}^{2}{p}^{2}+{\left(ed{E}_{z}^{{\rm{eff}}}-\eta {\lambda }_{{\rm{SO}}}\right)}^{2}}$, giving rise to a gap

7 $${\Delta }_{0}=| 2ed{E}_{z}^{{\rm{eff}}}-2{\lambda }_{{\rm{SO}}}| ,$$

which closes at the critical fields $ed({E}_{c}^{{\rm{eff}}})=\pm {\lambda }_{{\rm{SO}}}$ marking the transition from the topological Quantum Spin Hall state ($| {E}_{z}^{{\rm{eff}}}| < | {E}_{c}^{{\rm{eff}}}|$) to the trivial insulating system ($| {E}_{z}^{{\rm{eff}}}| > | {E}_{c}^{{\rm{eff}}}|$). The electron-phonon coupling is introduced in the tight-binding model as the linear expansion of the nearest-neighbor hopping in terms of the bond length as described in ref. ^50^. It enters in the low-energy model as a gauge field, allowing to replace derivatives with respect to the ionic displacement with derivatives with respect to the electronic momentum. This allows to establish a direct connection between electronic topological properties and the anomalous component of Born effective charges. Indeed, these are expressed in terms of the topological invariant ${{\mathcal{Z}}}_{2}$ as^50^

8 $${Z}_{s,xx}^{* ,{\rm{an}}}=(1-| {{\mathcal{Z}}}_{2}| )\xi \cdot {l}_{s}{\rm{sgn}}({E}_{z}^{{\rm{eff}}})\,\frac{A}{\pi },$$

where $| {{\mathcal{Z}}}_{2}| =1$ in the topological phases and ${{\mathcal{Z}}}_{2}=0$ in the trivial ones, A is the unit cell area, and *ξ* is the electron-phonon coupling parameter (see “Methods” for numerical values). The above result (Eq. 8) is well understood via a low-energy expansion of the Kane–Mele model, where the Born effective charge is determined by the difference of the spin valley Chern numbers evaluated at the ***K*** and ${{\boldsymbol{K}}}^{{\prime} }$ points^50^.

### Germanene

**Fig. 1:**
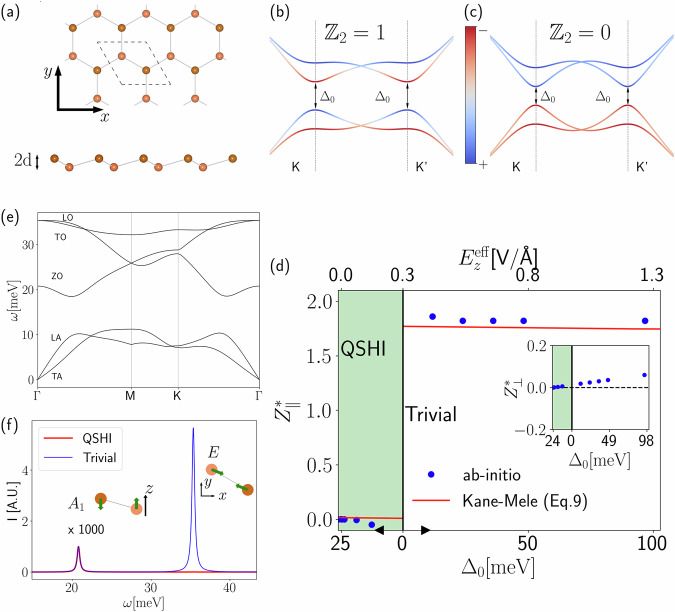
Germanene phonon infrared spectra. **a** Germanene buckled honeycomb structure, with the Cartesian reference which is chosen in this work. **b**, **c** The qualitative topological and trivial energetic band structure of the Kane–Mele model along the path $\Gamma \to K\to M\to {K}^{{\prime} }\to \Gamma$ (here identified by the projection over the sublattices (+, −)). The transition between the two phases is driven by the value of the external electric field ${E}_{z}^{{\rm{eff}}}$, the closing of the gap happening exactly at ${E}_{c}^{{\rm{eff}}}$. In (**d**), we show the in-plane (out-of-plane in the inset) values of Born effective charges, as computed using the Kane–Mele model via Eq. (8) or via ab initio calculations, as a function of the external electric field and the direct topological gap Δ_0_. Black arrows indicate the increase directions for Δ_0_ increase. Finally, **e** the ab initio phonon dispersion of the relevant modes at null electric field, while **f** the simulated infrared spectrum for the *A*_1_ and *E* modes, obtained from ab initio phonon frequencies and Born effective charges, while the linewidth is chosen for representability purposes, schematically represented as insets. The intensity of the *A*_1_ peak has been magnified by a factor of 1000 for presentability purposes.

Freestanding germanene displays a buckled honeycomb structure with $p\bar{3}m1$ layer group (crystallographic point group *D*_3*d*_), shown in Fig. 1a. Within our DFT calculations, the relaxed structure displays a lattice parameter of *a* = 4.05 Å, half buckling height of *d* = 0.34 Å, and a theoretical topological gap of Δ_0_ = 24.3 meV. Despite being in agreement with previous calculations^53,57,60,61^, the theoretical Δ_0_ is smaller than the experimental one, obtained for a sample grown on a GePt_2_ substrate, which has a lower bound of 70 meV^10^. Calculated phonon dispersion is shown in Fig. 1e. At *Γ*, there are two optical phonon modes that are Raman active but IR inactive: an out-of-plane *A*_1*g*_ mode at ~20 meV (ZO) and an in-plane *E*_*g*_ mode at 35 meV (LO/TO).

Germanium atoms form two inversion partner sublattices arising from Wyckoff position (WP) 2*d* in the unit cell. The local 3*m* site symmetries constraint the Born effective charge tensor to be diagonal, with two independent components ${Z}_{s,xx}^{* }={Z}_{s,yy}^{* }$ and ${Z}_{s,zz}^{* }$, while inversion symmetry implies that the effective charges of the two Ge atoms in the cell are the same, i.e., ${Z}_{s,xx}^{* }={Z}_{\parallel }^{* }$ and ${Z}_{s,zz}^{* }={Z}_{\perp }^{* }$. Since the charge-neutrality condition imposes $\sum _{s}{Z}_{s,\alpha \beta }^{* }=0$, both components ${Z}_{\parallel }^{* }$ and ${Z}_{\perp }^{* }$ are zero, consistently with the absence of IR-active modes.

An external electric field applied perpendicularly to the monolayer plane tunes a topological transition marked by the closure of the inverted gap at a critical field ${E}_{c}^{{\rm{eff}}}$, above which the trivial gap opens again, as depicted in Fig. 1b, c. Phonon frequencies are insensitive to the topological transition, and they are weakly modified by the applied field (see Section 5 SI (see Supplementary Material at URL will be inserted by publisher for additional details, which include refs. ^1,11,50,51,53–56^)). On the other hand, the orthogonal electric field breaks spatial inversion, reducing the symmetry to the *p*3*m*1 layer group (point group *C*_3*v*_). The Wyckoff position 2*d* is accordingly split to 1*b* + 1*c*, making the two Ge atoms inequivalent but preserving the local 3*m* site symmetries. It follows that Born effective charge components are allowed to acquire a non-zero value at finite applied fields, being opposite on inequivalent Ge atoms because of the charge-neutrality condition, ${Z}_{s,xx}^{* }={l}_{s}{Z}_{\parallel }^{* }$ and ${Z}_{s,zz}^{* }={l}_{s}{Z}_{\perp }^{* }$. Consistently, both optical modes become IR-active, and they are labeled as *A*_1_ and *E* irreducible representations of the *C*_3*v*_ point group. The evolution of effective-charge components calculated from first principles as a function of the applied field and across the topological transition is shown in Fig. 1d. The out-of-plane component ${Z}_{\perp }^{* }$ displays a continuous linear increase with the applied field, and it is insensitive to the topological transition, as shown in the inset of Fig. 1d. On the other hand, the in-plane ${Z}_{\parallel }^{* }$ component remains substantially zero in the QSHI phase, displaying only a slight deviation before undergoing a large enhancement at the critical field that induces the topological transition to the trivial phase. The topological origin of this jump can be appreciated by comparing the first principles result with the prediction, Eq. (8), of the Kane–Mele model for germanene, shown as a red line in Fig. 1d. The excellent agreement between first principles and model results can be attributed to the reliability of the low-energy Kane–Mele model in capturing the (dominant) topological contribution to the Born effective charges that arises from the neighborhoods of the Dirac points. Even though the ab initio bandgap is underestimated with respect to the experimental one by a factor of ~ 3, the simulation on stanene (Section 2 of SI (see Supplementary Material at URL will be inserted by publisher for additional details, which include refs. ^1,11,50,51,53–56^)), isostructural and isoelectronic to germanene but with a bandgap compatible with the experimental one of germanene, yields Born effective charges values close to the one of germanene. In general, analogous results, with the Born effective charges close to ~2, are obtained also in the trivial phases of the other Xenes compounds, such as silicene (Section 2 of SI (see Supplementary Material at URL will be inserted by publisher for additional details, which include refs. ^1,11,50,51,53–56^)) and gapped graphene^51^. In general, further trivial contributions from other electronic bands and regions of the Brillouin zone can be expected to affect quantitatively the total Born effective charges, as exemplified by jacutingaite discussed in the next section.

We are now in a position to assess the IR vibrational contribution to the optical conductivity of germanene. In Fig. 1f, we show the IR spectra obtained from ab initio phonon frequencies and Born effective charges for two values of the electric field ${E}_{z}^{1}=0.6{\rm{V/nm}}$ and ${E}_{z}^{2}=5.1\,{\rm{V}}/{\rm{nm}}$ corresponding to a gap of 19 meV and 24 meV, respectively, placing the system in the QSHI and trivial phase, respectively. In both cases, the vibrational IR spectrum is expected to display two peaks corresponding to the IR-active out-of-plane *A*_1_ and in-plane *E* modes. Since ${Z}_{\perp }^{* } < {Z}_{\parallel }^{* }$, we enhanced the *A*_1_ peak by a factor of 1000 for illustration purposes, and we normalized both spectra to such intensity. The (rescaled) two-peak structure is clearly visible in the trivial phase, where the *E* peak is the most prominent feature of the IR spectrum. Instead, the spectrum in the QSHI phase only displays the *A*_1_ peak, while the *E* peak practically disappears.

### Jacutingaite

**Fig. 2:**
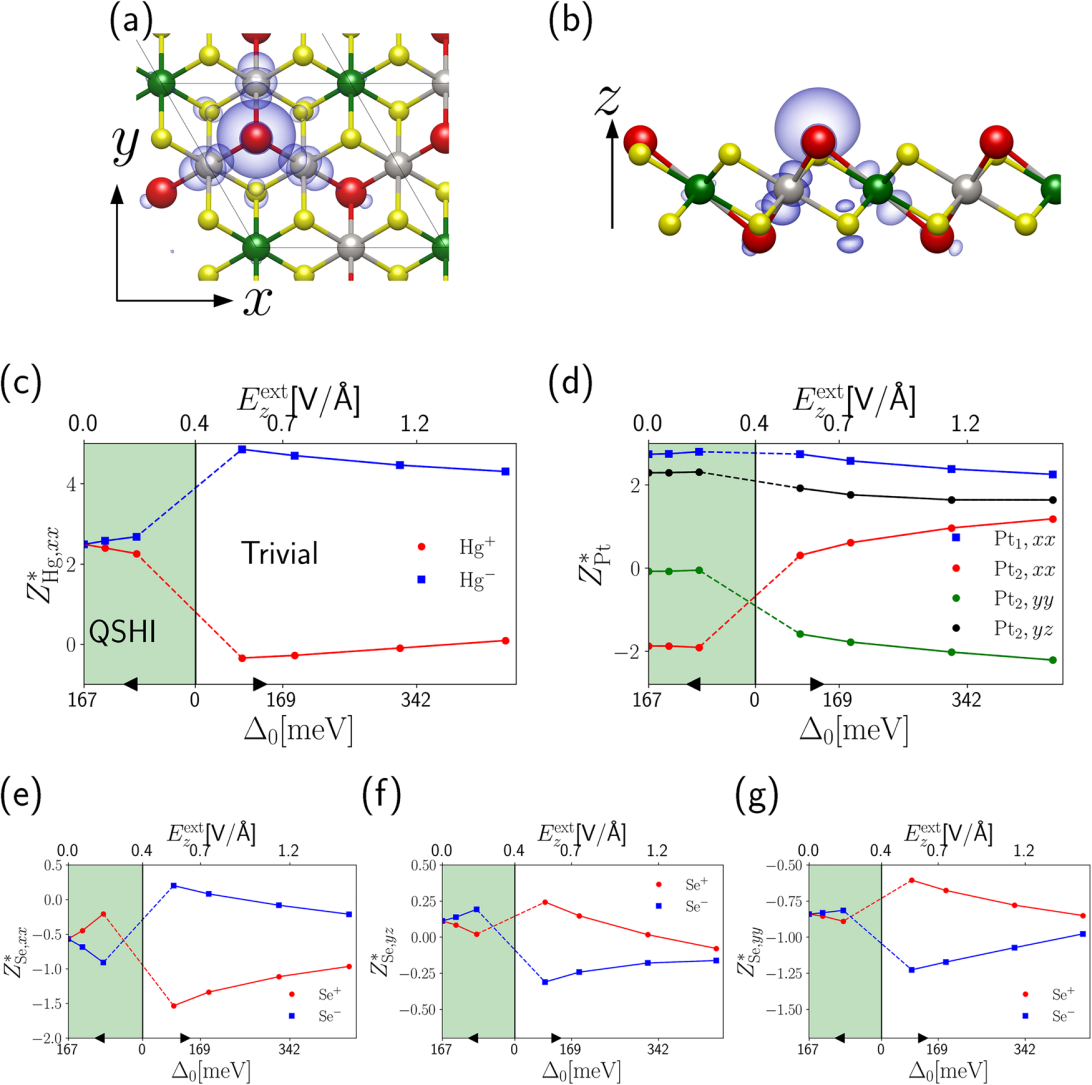
Jacutingaite Born effective charges. **a** Top and **b** side view of jacutingaite. The color code for the atoms is: Hg red, Pt_1_ green, Pt_2_ gray, Se yellow. We label the Hg atoms that are towards more positive(negative) *z* as Hg^+^(Hg^−^), and the same for Se^+^(Se^−^). Maximally localized Wannier functions built from the low-energy spectrum are represented as isocontours. In the panels below, we plot relevant components of Born effective charges as a function of the direct topological gap at *K* point Δ_0_, varied by applying an external orthogonal electric field, for **c** Hg^±^, **d** Pt_2_, **e**–**g** Se^±^. Black arrows indicate increasing directions for Δ_0_. As described in the text, Hg^±^ strongly resemble the results of the Kane–Mele model, as per Eq. (9). On the contrary, the behavior of Se^±^ and Pt_2_ atoms is more complicated, even if jumps (or, rather, component exchanges) are still rationalized in terms of the topological transition, as described in the text. Dashed lines across the transition are intended as a guide to the eye, whereas the continuous ones describe the trends in each phase.

The monolayer of jacutingaite (Pt_2_HgSe_3_) is a QSHI with a large indirect bandgap between the K and M points of ~0.15 eV, and a comparable topological direct gap of ~0.17 eV at the K point^21^. Its structure belongs to the same layer group $p\bar{3}m1$ of germanene or buckled graphene, the Hg atoms occupying the same 2*d* Wyckoff positions of Ge in the buckled honeycomb lattice [Fig. 2a, b]. The low-energy physics of the topological gap is well described by an effective two-band model analogous to a Kane–Mele model^21^, that can be constructed in a basis of maximally localized Wannier functions (MLWFs)^62^. The MLWFs contributing to the low-energy effective model are centered on Hg atoms, i.e., on the sites of the buckled honeycomb lattice, and they display a dominant Hg *s* character, with weaker but non-negligible hybridization with nearest-neighbor Pt atoms bridging Hg [gray-colored in Fig. 2a, b] and with surrounding Se atoms. Given the stringent analogy with germanene, the topological phases of jacutingaite can be tuned by means of an external orthogonal electric field, which also, in this case, lowers the symmetry of the system to *p*3*m*1. Different from germanene, the electric field induces a strong rearrangement of the ionic positions inside the unit cell. The ionic distortion is responsible for a relevant reduction of the critical field for the topological transition and, as discussed in ref. ^21^, it represents by itself a mechanism to drive the system to the trivial state.

Being described by an effective Kane–Mele model, the contributions to the in-plane Born effective charges coming from the low-energy sector of the Hamiltonian, and therefore due to the orbitals entering in the topological band inversion mechanism, are expected to present a discontinuous jump across the topological phase transition. The number of independent components of the Born effective charge tensors is determined by the site symmetry of the representative of each inequivalent atomic species, as discussed in the “Methods” section. In Fig. 2, we consider the only independent in-plane component of the diagonal Born effective charges tensor of Hg and Pt_1_ and the non-zero in-plane components of the representative Pt_2_ and Se atoms for which the twofold rotation axis is parallel to *x*.

When the symmetry is lowered to *p*3*m*1, previously inversion-related Hg and Se atoms become inequivalent. We will distinguish such atoms with the labels Hg^±^ and Se^±^, where the + means that the atom is above the plane of the system and accordingly for the −, as shown in Fig. 2b. On the other hand, inversion-symmetry breaking does not modify the local site symmetries and the form of the Born effective charges tensors on all atoms, though the values of the components for Hg^+^ (Se^+^) and Hg^−^ (Se^−^) will now be different.

The Born effective charges computed ab initio and the evolution through the topological transition of selected components are displayed in Fig. 2. Coherently with the above analysis, the in-plane component of the Born effective charge for Hg experiences a discontinuous jump across the topological transition, as shown in Fig. 2c. For Pt_1_, not taking part in the Kane–Mele effective model, no such discontinuity appears [Fig. 2d]. Interestingly, some components of the effective charges of Pt_2_ and Se also display discontinuities, shown in Fig. 2e–g.

Notice that, differently from germanene, Born effective charges are not zero at null external field. This is because the low-energy sector of the electronic spectrum only contributes to a portion of the Born effective charges value, and therefore only such contribution displays a relevant jump associated with the topological phase transition, while the remainder has a trivial (and negligible in the first instance) behavior with the applied field. In other words, the ab initio behavior of Born effective charges of jacutingaite as a function of ${E}_{z}^{{\rm{eff}}}$ in the studied range can be rationalized via the following expression:

9 $${Z}_{s,\alpha \beta }^{* }({E}_{z}^{{\rm{eff}}}) \sim {Z}_{s,\alpha \beta }^{* }(0)+\delta {Z}_{s,\alpha \beta }^{* }\theta ({E}_{z}^{{\rm{eff}}}-{E}_{c}),$$

where $\delta {Z}_{s,\alpha \beta }^{* }$ is the topological jump, different for every atom and component, with the only general rule that $\delta {Z}_{s,z\alpha }^{* }=0$ where *α* = *x*, *y*, *z* since out-of-plane induced polarization is not related to electronic topological effects. For example, $\delta {Z}_{{{\rm{Hg}}}^{\pm },xx}^{* }$ clearly follows the behavior of Eq. (8), with *l*_*s*_ = ±1 referring to the two inequivalent Hg^±^ atoms, as expected from the dominant mercury character of the MLWFs realizing the effective Kane–Mele model. In particular, the magnitude of the discontinuous jump for Hg^±^ is $| \delta {Z}_{{{\rm{Hg}}}^{\pm },xx}^{* }| \sim 2.5$.

For Se^±^ and Pt_2_, the situation is different. In the first case, across the topological transition, there is an apparent exchange of components between the partners Se^±^. Concerning Pt_2_, where some components display an almost trivial behavior while others display large jumps, an analogous exchange of contributions seems to happen, but for different Cartesian components on the same atom. Both behaviors are reasonably traceable to the exchange of conduction and valence wavefunction occurring at the topological phase transition rather than a jump in the form of Eq. (8). In fact, Se^±^ and Pt_2_ orbitals enter in the determination of MLWFs, but in a weaker fashion with respect to Hg. Therefore, the proper topological jump of Eq. (8) is expected to be much weaker. Nonetheless, across the topological transition, some components of Se^±^ and Pt_2_ orbitals are energetically swapped between valence and conduction bands, as well as their contribution to effective charges via the exchange of electronic wavefunctions, ending up in exchanges of components. Finally, we notice that the trivial behavior of Se effective charges under an electric field is not fully negligible as for Eq. (9), and discussed in Section 3 of the SI (see Supplementary Material at URL will be inserted by publisher for additional details, which include refs. ^1,11,50,51,53–56^).

We turn now to analyze the vibrational IR spectrum of jacutingaite, focusing on in-plane phonon modes that are expected to display signatures of the topological transition. Even in the absence of an applied field, among the 33 optical modes at *Γ* listed in Supplementary Table 5 (see Supplementary Material at URL will be inserted by publisher for additional details, which include refs. ^1,11,50,51,53–56^), seven in-plane phonons belong to the IR-active *E*_*u*_ representation, consistently with the non-zero Born effective charges in the $p\bar{3}m1$ phase of jacutingaite. Four other optically active in-plane *E*_*g*_ modes are instead Raman active, but they can show IR activity when the inversion symmetry is broken by the external electric field. The frequencies are quite close to the one of the bulk sample, where the ab initio computation is in good agreement with Raman experiments^63–65^. Although phonon frequencies show a weak dependence on the applied field (see Section 4 of SI (see Supplementary Material at URL will be inserted by publisher for additional details, which include refs. ^1,11,50,51,53–56^)), the strength of their coupling with IR radiation is significantly affected by the topology of the underlying electronic structure.

**Fig. 3:**
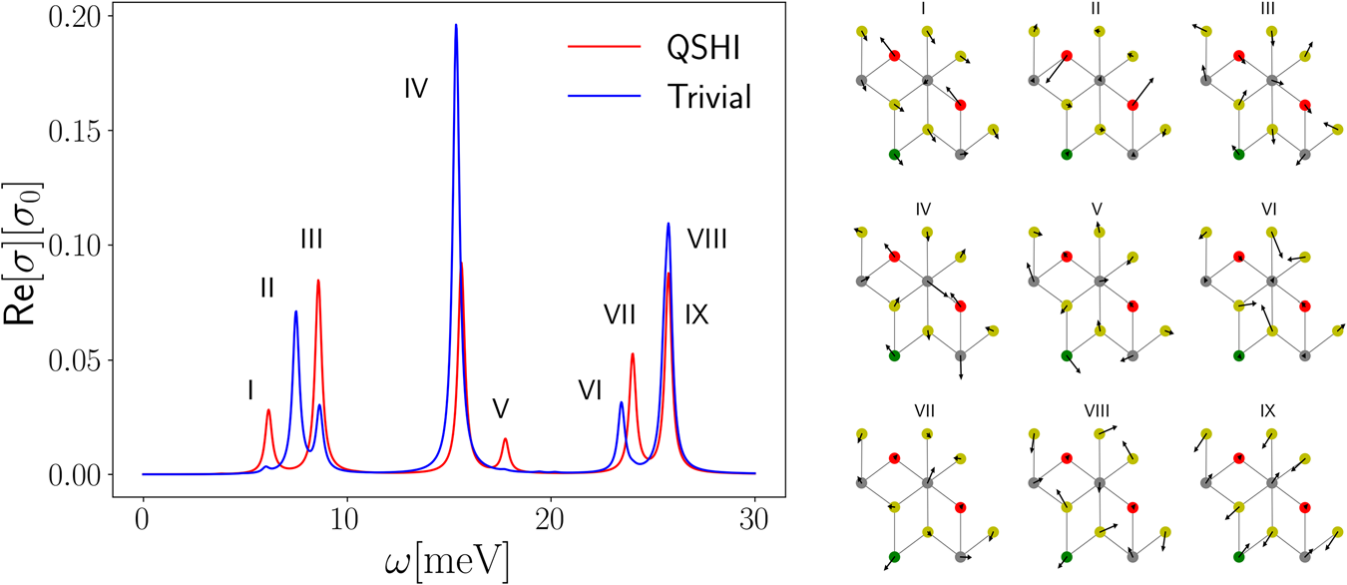
Jacutingaite phonon infrared spectra. Left panel: infrared optical spectrum of jacutingaite, for both the QSHI and the trivial phases, for gaps at the ***K*** point of Δ_1_ = 0.15 eV (${E}_{z}^{{\rm{ext}}}=0$ V/Å) and Δ_2_ = 0.18 eV (${E}_{z}^{{\rm{ext}}}=0.74$ V/Å).The linewidth *γ*_*ν*_ = 0.4 meV is chosen equal for all modes for representation purposes. Nine distinct phonons are identified and labeled with Roman numerals; the corresponding in-plane projections of the atomic displacements are represented in the right panel (the color code for atomic species is the same as for Fig. 2). As discussed in the text, the mode II is the clear analog of the *E* mode of germanene, with the Hg^±^ atoms playing the same role of the Ge one. The behavior of the other modes is explained in the text.

The computed spectrum at finite fields for the QSHI phase with a topological gap of Δ_1_ = 0.15 eV is compared in Fig. 3 with that of the trivial phase with a comparable gap Δ_2_ = 0.18 eV. Out of the 11 IR-active modes, only nine are found to significantly contribute to the optical response, which we label by Roman numerals. The most interesting ones are the vibrational modes II and VI, both deriving from the *E*_*g*_ Raman-active modes of inversion-symmetric jacutingaite at zero field. Mode II is due to Hg atoms moving in opposite phase, and it is the direct analog of the *E*_*g*_ → *E* mode of germanene, coherently being infrared inactive in the absence of an external field and displaying a huge intensity jump across the topological phase transition. The behavior of mode VI can be similarly understood as due to the out-of-phase in-plane motion of Se atoms, giving rise to a negligible though finite IR intensity that undergoes a sizeable enhancement through the topological transition. Finally, the intensity of the seven modes that are IR-active at zero field changes relevantly as the system evolves from the QSHI to the trivial phase. Among these, only mode I is significantly contributed by Hg atoms, that move in phase and with the same amplitude in absence of the field (see Fig. 3); with increasing field, a growing unbalance between the Hg^±^ atomic displacements enhances the dependence on the topological contribution $\delta {Z}_{{{\rm{Hg}}}^{\pm },xx}$ of the related oscillator strengths, ultimately leading to a discontinuous drop of its IR intensity at the topological transition. Similar drops are observed for modes III, V, and VII, while mode IV and quasi-degenerate modes VIII, IX display increasing intensity in the trivial phase. Despite displaying distinct and detectable IR spectral features related to the topological phase transition, all such changes are contributed mostly by Pt atoms, and cannot thus be rationalized within the results of the effective Kane–Mele model.

### Dynamical effects

If the topological gap Δ_0_ is of the order of magnitude of a relevant infrared active phonon frequency *ω*_ph_, the phonon frequency become resonant with electronic excitations and therefore the Born effective charges cannot any more be approximated by their static value. This is for example the case for germanene, if the gap is taken as its theoretical DFT value. Nonetheless, also for large topological gap insulators such as jacutingaite, there will always be a regime where Δ_0_ ~ *ω*_ph_ near the topological transition. In this case, the vibrational IR response of the system is quantified by the complex valued frequency dependent dynamical effective charges^51,52^. The interplay between the electronic and phonon excitation produces frequency dependent Fano lineshapes on top of the electronic excitation spectrum in the optical spectra, rather than off-resonant Lorentzian lineshapes, which qualitatively and quantitatively change the shape of the infrared spectrum, as predicted and seen experimentally in graphene systems^66^.

Since dynamical effects are relevant when the electronic gap is small, they are well described by the low-energy Kane–Mele model. The reliability of the model is further verified by comparing the Kane–Mele and the ab initio static electric susceptibility reflecting the effect of electronic excitations (shown in Supplementary Fig. 12). It is then easy to obtain analytical expressions for *σ*^el^(*ω*), as well as for the diagonal in-plane-components ${Z}_{s,xx}^{* }({\omega }_{{\rm{ph}}})$, as shown in the Section 5 of the SI (see Supplementary Material at URL will be inserted by publisher for additional details, which include refs. ^1,11,50,51,53–56^). Notice that the relation ${Z}_{s,xx}^{* }({\omega }_{{\rm{ph}}})={l}_{s}{Z}^{* }({\omega }_{{\rm{ph}}})$ still holds even in the presence of dynamical effects if, as assumed in this work following ref. ^50^, the electron-phonon coupling depends only on the relative distance between two atoms. The conductivity tensor is also diagonal for $p\bar{3}m1$ and *p*3*m*1 symmetries, with *σ*_*xx*_ = *σ*_*yy*_; in the following we refer to the in-plane component as *σ*.

We now sum up the most important results. Defining *z* = (*ω*−*ω*_ph_)/*γ*_ph_, the ionic contribution to the optical conductivity is described by a Fano function^67^

10 $${\sigma }^{{\rm{ion}}}(\omega )=P\frac{{q}^{2}-1+2qz}{(1+{q}^{2})(1+{z}^{2})}+iP\frac{({q}^{2}-1)z-2q}{({z}^{2}+1)(1+{q}^{2})},$$

where

11 $$P=\frac{{\sigma }_{0}\bar{\omega }}{{\gamma }_{{\rm{ph}}}}| Z({\omega }_{{\rm{ph}}}){| }^{2},\quad q=-\frac{\,\text{Re}\,[{Z}^{* }({\omega }_{{\rm{ph}}})]}{\,\text{Im}\,[{Z}^{* }({\omega }_{{\rm{ph}}})]},$$

are the phonon strength and the Fano asymmetry parameters, respectively. Depending on the asymmetry parameter, the Fano function can describe a variety of different profiles ranging from a positive Lorentzian peak (*q* → −*∞*, when ${\rm{Im}}{Z}^{* }\to 0$) to highly antisymmetric dispersive profiles (*q* → −1) and even negative peaks (*q* = 0)^66^. For germanene, $\hslash \bar{\omega }=\frac{8{\hslash }^{2}}{{M}_{{\rm{Ge}}}A}=0.032\,{\rm{meV}}$, while ${\sigma }_{0}=\frac{\pi {e}^{2}}{2h}$ is the universal conductivity, corresponding to the one of clean graphene. The effects of the electric field on *ω*_ph_ are taken into account by computing the variation of the static frequency from ab initio calculations, while dynamical effects on the phonon frequency are computed analytically within the low-energy model (see Section 5 SI (see Supplementary Material at URL will be inserted by publisher for additional details, which include refs. ^1,11,50,51,53–56^)). The latter are small compared with the first ones, causing only a slightly enhancement at the resonance between the phonon frequency and the gap. *γ*_ph_ = *γ*_ph-ph_ + *γ*_e-ph_ is the full width at half maximum determined by the phonon-phonon scattering, taken from experimental measures on bulk Germanium and assumed independent from electronic resonances *γ*_ph-ph_ = 0.07 meV^68^, and the electron-phonon coupling contribution *γ*_e-ph_, computed including dynamical effects in the Kane–Mele model as discussed in Section 5 of the SI (see Supplementary Material at URL will be inserted by publisher for additional details, which include refs. ^1,11,50,51,53–56^). The phonon linewidth is significantly enhanced by the electronic resonance, almost doubling its value.

**Fig. 4:**
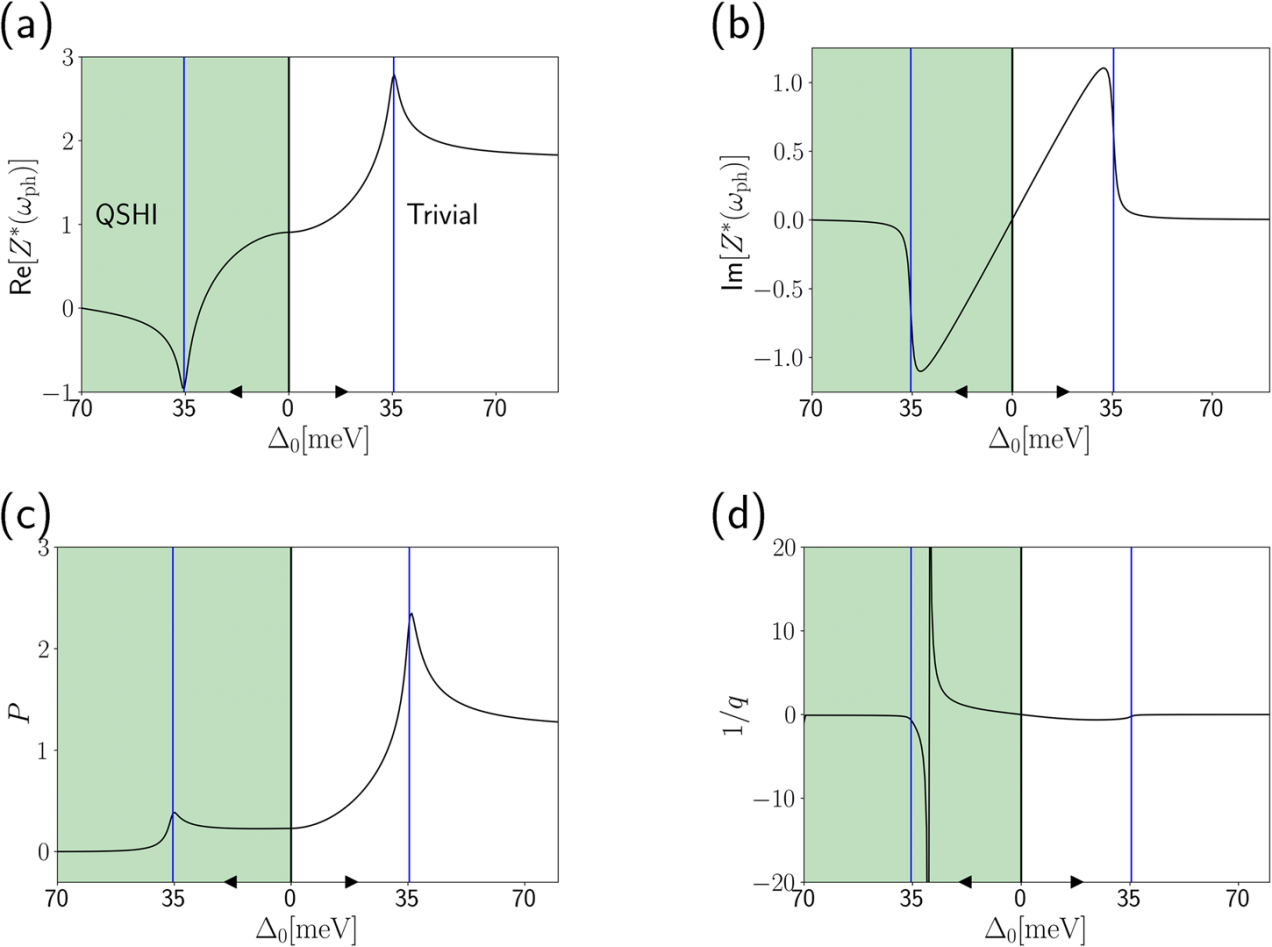
Dynamical parameters for germanene optical conductivity. **a** Real and **b** imaginary parts of the dynamical Born effective charges computed at the phonon frequency *ω*_ph_ = 35.5 meV for the Kane–Mele model across the QSHI and trivial phases as a function of the direct gap at *K* point Δ_0_ varied applying the external orthogonal electric field. The topological unperturbed gap is taken from the one experimental germanene 70 meV, whereas the adiabatic phonon frequency of *ω*_ph_ = 35.5 meV, as well as the electron-phonon coupling parameters are predicted by ab initio calculations. The related phonon strength and Fano asymmetry parameters for the ionic conductivity are represented in (**c**, **d**). Black arrows indicate increase directions for Δ_0_ while the vertical blue lines correspond to the adiabatic phonon frequency energy marking the condition Δ_0_ = *ω*_ph_.

The complex valued dynamical effective charges, the phonon strength *P* and the Fano asymmetry *q* parameters are plotted in Fig. 4 for a Kane–Mele model with Δ_0_ = 70 meV at null external field, as compatible with the experimentally measured one in germanene^10^, and a adiabatic phonon frequency of *ω*_ph_ = 35.5 meV at null external field as predicted by ab initio calculations. The topological transition affects the Born effective charges and the parameters *P* and *q* in a different way with respect to the static case. Most notably, the discontinuous jumps of Eq. (8) are smoothed by dynamical effects, and Born effective charges acquire a relevant imaginary part in the resonant condition with an opposite sign in the two topological phases.

**Fig. 5:**
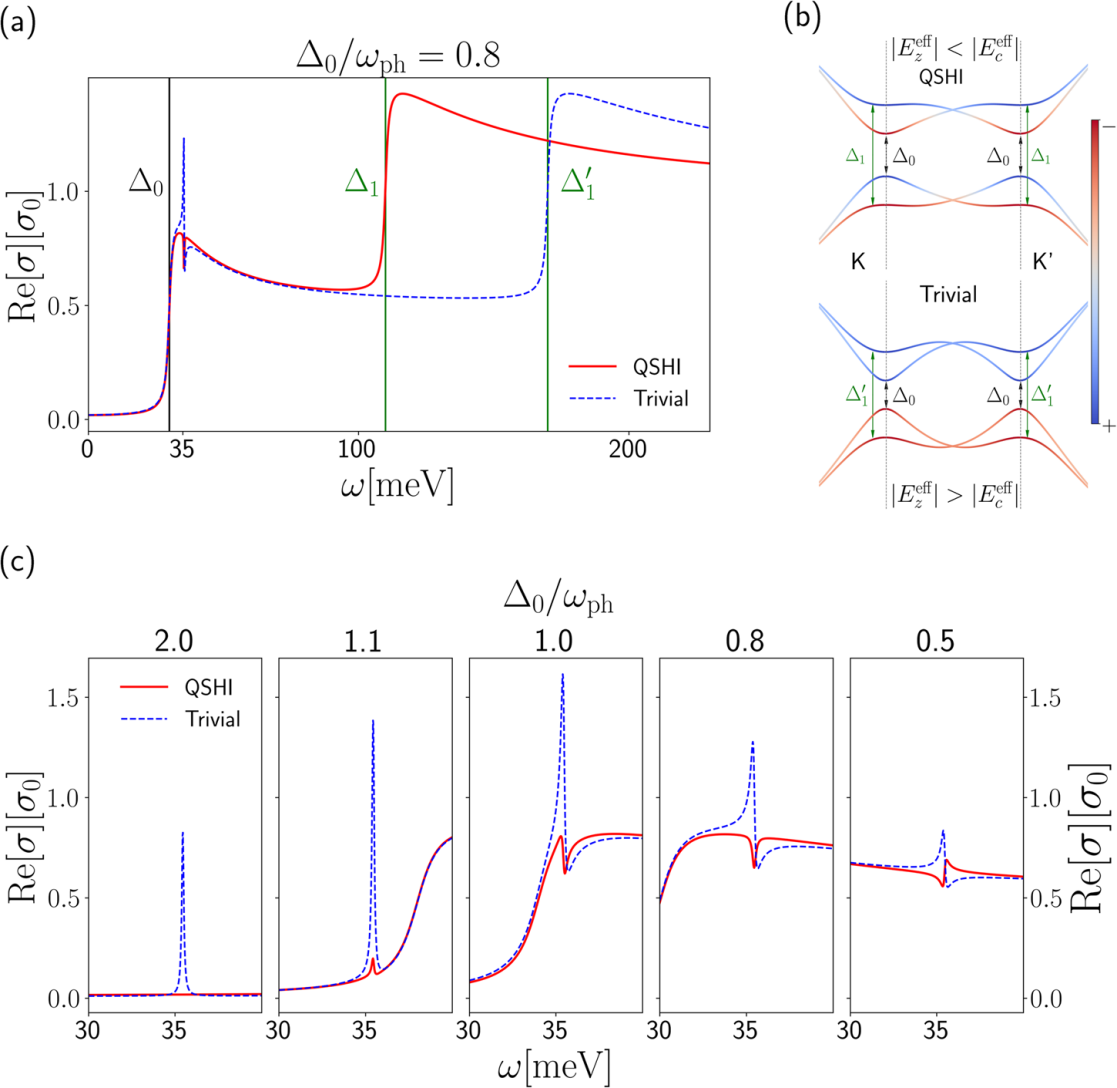
Optical conductivity of germanene with dynamical effects. **a** Real part of the optical conductivity of the Kane–Mele model, taking into account dynamical effects on both the electronic and ionic contributions, in the QSHI and the trivial phases for the same direct gap Δ_0_ = 0.8*ω*_ph_, where the adiabatic phonon frequency of *ω*_ph_ = 35.5 meV is obtained from ab initio calculation. The relevant electronic transitions marked by the vertical black (direct gap transition between bands undergoing topological band inversion) and green lines (transition between the higher and lower energy bands, not affected by topological effects) are described in the qualitative energy bands of the Kane–Mele model (here identified by the projection over the sublattices (+, −)) in (**b**). The conductivity is expressed in units of the universal conductivity *σ*_0_ = *e*^2^/4*ℏ*. **c** Ionic contribution to the real part of the optical conductivity as a function of the ratio Δ_0_/*ω*_ph_, where the direct gap at the *K* point is varied by mean of an external orthogonal electric field. Where the topological gap is much larger than the phonon frequencies, we recover the same results as for the static treatment (i.e., disappearence of the phonon feature in the QSHI phase). When the topological gap is comparable or smaller than the phonon frequencies, dynamical effects kick in, and the phonon is visible even in the QSHI phase. Nonetheless, the two phases are still very clearly distinguishable due to the different shape of the Fano profile.

We report in Fig. 5a the real part of the optical conductivity including both electronic and phonon features (see Eq. 1), where the relevant electronic transitions are depicted in Fig. 5b. We also report the ionic contribution to the real part of the optical conductivity, in Fig. 5c, for several values of Δ_0_/*ω*_ph_. In the off-resonance condition Δ_0_/*ω*_ph_ ~ 2.0, the phonon spectral feature is visible only in the trivial phase while vanishing in the topological one, as already discussed in the section “Germanene.” Approaching the resonance Δ_0_/*ω*_ph_ ~ 1, the phonon contribution becomes non vanishing also in the QSHI phase but with markedly different spectral features from the trivial phase. For phonon energies larger than the gap, the Fano lineshapes describing phonon resonances between the two phases differ qualitatively, going from positive to negative peaks or having opposite dispersive behavior. Even though the intensity difference becomes not so large as in the off-resonant condition, the lineshape corresponding to different topological phases with the same gap are clearly discernible. The transmittance and the reflectivity spectra of germanene, reported in Supplementary Section 5, as well as the Born effective charges in the trivial state report expected signals comparable to the one of graphene and its heterostructure^54,55,66,69,70^.

## Discussion

In this work, we showed that infrared response can be used as a marker to characterize topological transitions on promising and already experimentally studied materials, such as germanene and jacutingaite. We showed via first principles calculations that the Born effective charges display sudden finite jumps across the topological phase transitions, in agreement with predictions based on a low-energy Kane–Mele model.Extrinsic effects (such as strain or alloying) may quantitatively impact the value of the Born effective charges^48,51,71^, but they are not expected to display quantized jumps across the topological transition. Importantly, such jumps are very large, of order 2 for germanene and up to 2.5 for the Hg atoms of jacutingaite, causing a clear cut change in the infrared vibrational spectrum across the transition. While in germanene the vibrational resonances almost disappear, in jacutingaite large intensities are preserved in the markedly different spectral profiles due to the different topological states, a change that can be detected by experimental measures with the same sensitivity. We also showed that our conclusions are robust with respect to dynamical mechanisms, that become relevant when the energy gap is of the same order of the frequency of the infrared active phonon. Our results pave the way for the determination of the topological properties of materials via the use of optical spectroscopy techniques in two-dimensional system. In light of the giant enhancement of Born effective charges in 1D systems^72^, we speculate that the results of this work may be extended to study the transition from 2D to 1D topological systems^73^.

## Methods

### Ab initio parameters

First principles calculations were performed using Quantum ESPRESSO^74^ with Perdew–Burke–Ernzerhof (GGA) exchange functional^75^ using Pseudo Dojo full relativistic Optimized norm-conserving Vanderbilt pseudo-potentials^76,77^ with a plane wave cutoff on the wavefunctions of 80 Ry for germanene, stanene and silicene and 60 Ry for jacutingaite. The convergence threshold for the phonon calculation is set at 10^−18^ (corresponding to the input word tr2_ph in Quantum Espresso). Since germanene, stanene and silicene have a small gap, Brillouin zone sampling is carried out using a telescopic grid with an elevated density around the K and ${{\rm{K}}}^{{\prime} }$ points to achieve convergence, according to the procedure described in Appendix B of ref. ^78^ with the following parameters: for germanene and stanene ${\mathcal{N}}=50,\,l=5,\,L=9,\,p=8$ corresponding to 481 irreducible points, for silicene ${\mathcal{N}}=50,\,l=11,\,L=6,\,p=8$ having 3386 irreducible points. For jacutingaite, by virtue of the larger gap, an 18 × 18 × 1 Monkhorst-Pack mesh^79^ is sufficient to achieve convergence. An interlayer distance larger than 19 Å is used to separate the monolayers of germanene, stanene and silicene and of 30 Å for the jacutingaite monolayer. The 2 × 2 MLWF Hamiltonian describing the valence and conduction bands of jacutingaite is performed using WANNIER90^80^. In all our calculation we implement the 2D cutoff technique with the electric field introduced in field-effect transistor setup, in the double gate configuration^81^. The charged plates are placed symmetric with respect to the plane of the 2D system at *z* = ±0.221 in crystalline units in all the studied systems. The generated capacitor-like potential increases (or decreases) linearly until the potential barriers of height *E* = 34 eV placed at *z* = ±0.22 in crystalline units. All the structures are relaxed with a threshold of at least 7 ⋅ 10^−7^ eV for the energy and 7 ⋅ 10^−6^ eV/Å for the forces.

### Low-energy model parameters

The model parameters of germanene, as well as for stanene and silicene, are obtained from the ab initio simulations. We use Eq. (7) to first obtain *λ*_SO_ from the value of the ab initio topological gap at null electric field. Then, we apply a finite external electric field to the system with the methodology explained in the previous paragraph, and use (7) to deduce the total effective field ${E}_{z}^{{\rm{eff}}}$ associated to the ab initio energy gap that arises at such given external electric field.

The electron-phonon coupling is computed in absence of electric-field, with the same grids used for the Born Effective charges calculation. In detail, we compute $\langle {g}_{{\boldsymbol{\Gamma }}}^{2}\rangle =\left.\sum _{i,j=c,v}\right\vert \left\langle {\psi }_{i{\bf{K}}}\right\vert \frac{\partial H}{\partial {{\bf{u}}}_{\Gamma ,\nu }}\left\vert {\psi }_{j{\bf{K}}}\right\rangle \cdot {{\bf{e}}}^{\nu }/{\left.\sqrt{2M{\omega }_{\Gamma ,\nu }/\hslash }\right\vert }^{2}/4$, where *ψ*_*i*/*j*,**K**_ are the valence/conduction (*v*/*c*) Bloch functions at the K point, and we then mediate over the degenerate modes *ν* = TO/LO as well^82^. The parameter used in the tight-binding method are found by $\xi =\frac{1}{{b}_{0}{t}_{1}}\sqrt{\frac{4\langle {D}_{{\boldsymbol{\Gamma }}}^{2}\rangle }{9}}$, where $\langle {D}_{{\boldsymbol{\Gamma }}}^{2}\rangle =\langle {g}_{{\boldsymbol{\Gamma }}}^{2}\rangle (2M{\omega }_{\Gamma }/\hslash )$ and *t*_1_ is fitted from the first principles bands and *b*_0_ is the nearest-neighbor distance, as detailed in refs. ^51,83^. For germanene, we obtain $\xi =0.394{\frac{1}{\text{\AA}}}^{2}$.

### Optical conductivity

The detailed calculation of the optical conductivity is explained in the supplementary material. We here give a brief summary of the procedure. The low-energy approximation describes properly system with a sufficient small gap, as for the ones considered in this work. In the approximations of this work, the Kane–Mele model reduces to twotime-reversal-related copies of the Haldane model, one for each spin. The low-energy approximation of the Haldane model allows to compute analytically the response functions even at finite frequency *ω* and electronic lifetime *η*. The procedure is to express the conductivity is its linear response form

12 $$\begin{array}{l}{\sigma }_{\alpha \alpha }(\omega )={\sigma }_{0}\frac{i}{{N}_{{\boldsymbol{k}}}A}\sum _{{\boldsymbol{k}},ij}\frac{{f}_{{\boldsymbol{k}},i}-{f}_{{\boldsymbol{k}},j}}{{\epsilon }_{{\boldsymbol{k}},i}-{\epsilon }_{{\boldsymbol{k}},j}+\hslash \omega +i\zeta }\\\qquad\langle {u}_{{\boldsymbol{k}},i}| \frac{\hslash {v}_{\alpha }}{{\epsilon }_{{\boldsymbol{k}},i}-{\epsilon }_{{\boldsymbol{k}},j}}| {u}_{{\boldsymbol{k}},j}\rangle \langle {u}_{{\boldsymbol{k}},j}| \hslash {v}_{\alpha }| {u}_{{\boldsymbol{k}},i}\rangle\end{array}$$

where ${\sigma }_{0}=\frac{{e}^{2}}{4\hslash }$ is the universal conductivity of clean graphene, *f* are the Fermi–Dirac statistical occupation factor, *u* are the periodic part of the Bloch functions, *ϵ* the band energies, and *v* is the velocity operator. Then, one can pass from the reciprocal space quasi-momentum to the energy variable, specifying all quantities of Eq. (12) for the model. Finally, one can correct the electronic conductivity of the model, by matching the static value of its imaginary part to the one computed via ab initio calculations, that takes into account all the bands of the material and not only the one of the model. We checked that this effect is irrelevant for the purposes of this work, as shown in the supplementary information.

### Symmetries of jacutingaite atoms

Hg atoms occupy the 2*d* Wyckoff Position (WP), like the Ge atoms in germanene, so the symmetries constrain the corresponding Born effective charge tensors to be diagonal with two independent components that are equal on equivalent Hg atoms in the $p\bar{3}m1$ phase, being related by inversion symmetry. Pt atoms occupy two different WPs, 1*a* and 3*e*. The 1*a* platinum sitting at the center of each honeycomb, henceforth labeled as Pt_1_, displays the full crystallographic point-group symmetry *D*_3*d*_ ($\bar{3}m$) and a diagonal charge tensor with equal in-plane components. The 3*e* Pt atoms, which contribute to the MLWFs realizing the Kane–Mele effective model and henceforth labeled as Pt_2_, display a 2/*m* site symmetry, being related by the threefold rotation about the out-of-plane direction. Finally, Se atoms occupy the 6*i* WP, with *m* site symmetry and being related by inversion and threefold rotation symmetries. The Born effective charge tensor has 5 independent components for both Pt_2_ and Se atoms. Considering the Pt_2_ and Se atoms for which the twofold rotation axis is parallel to *x*, site symmetries impose ${Z}_{{{\rm{Pt}}}_{2}/{\rm{Se}},xy}^{* }={Z}_{{{\rm{Pt}}}_{2}/{\rm{Se}},xz}^{* }={Z}_{{{\rm{Pt}}}_{2}/{\rm{Se}},zx}^{* }={Z}_{{{\rm{Pt}}}_{2}/{\rm{Se}},yx}^{* }=0$.

## Supplementary information

Supplementary Information

## Data Availability

The original data are available from the corresponding author upon reasonable request.

## References

[1] Kane, C. L. & Mele, E. J. Quantum Spin Hall effect in graphene. *Phys. Rev. Lett.* **95**, 226801 (2005). 16384250 10.1103/PhysRevLett.95.226801

[2] Vanderbilt, D. *Berry Phases in Electronic Structure Theory: Electric Polarization, Orbital Magnetization and Topological Insulators* (Cambridge University Press, 2018).

[3] Hasan, M. Z. & Kane, C. L. Colloquium: topological insulators. *Rev. Mod. Phys.* **82**, 3045–3067 (2010).

[4] Bernevig, B. A. & Hughes, T. L. *Topological Insulators and Topological Superconductors* (Princeton University Press, 2013).

[5] Marrazzo, A., Gibertini, M., Campi, D., Mounet, N. & Marzari, N. Relative abundance of z2 topological order in exfoliable two-dimensional insulators. *Nano Lett.* **19**, 8431–8440 (2019). 31658415 10.1021/acs.nanolett.9b02689

[6] Grassano, D., Campi, D., Marrazzo, A. & Marzari, N. Complementary screening for quantum spin Hall insulators in two-dimensional exfoliable materials. *Phys. Rev. Mater.* **7**, 094202 (2023).

[7] Gilbert, M. J. Topological electronics. *Commun. Phys.* **4**, 70 (2021).

[8] Lodge, M. S., Yang, S. A., Mukherjee, S. & Weber, B. Atomically thin quantum spin Hall insulators. *Adv. Mater.* **33**, 2008029 (2021). 10.1002/adma.202008029 33893669

[9] Weber, B. et al. 2024 roadmap on 2D topological insulators. *J. Phys. Mater.* **7**, 022501 (2024).

[10] Bampoulis, P. et al. Quantum spin Hall states and topological phase transition in germanene. *Phys. Rev. Lett.* **130**, 196401 (2023). 37243643 10.1103/PhysRevLett.130.196401

[11] Ezawa, M. A topological insulator and helical zero mode in silicene under an inhomogeneous electric field. *New J. Phys.* **14**, 033003 (2012).

[12] Klaassen, D. J., Boutis, I., Castenmiller, C. & Bampoulis, P. Tunability of topological edge states in germanene at room temperature. *J. Mater. Chem. C* **12**, 15975–15980 (2024). 10.1039/d4tc02367f PMC11382626 39262567

[13] Zandvliet, H. J. W., Klaassen, D. J. & Bampoulis, P. Evidence for a different dispersion of the topological edge state of germanene at armchair and zigzag edges. *Phys. Rev. B* **109**, 115419 (2024).

[14] Collins, J. L. et al. Electric-field-tuned topological phase transition in ultrathin Na3Bi. *Nature* **564**, 390–394 (2018). 30532002 10.1038/s41586-018-0788-5

[15] Nadeem, M., Di Bernardo, I., Wang, X., Fuhrer, M. S. & Culcer, D. Overcoming Boltzmann’s tyranny in a transistor via the topological quantum field effect. *Nano Lett.* **21**, 3155–3161 (2021). 33780625 10.1021/acs.nanolett.1c00378

[16] Cabral, A. R., Galbiatti, H. F., Kwitko-Ribeiro, R. & Lehmann, B. Platinum enrichment at low temperatures and related microstructures, with examples of hongshiite (PtCu) and empirical ‘Pt_2_HgSe_3_’ from Itabira, Minas Gerais, Brazil. *Terra Nova* **20**, 32–37 (2008).

[17] Vymazalova, A. et al. Jacutingaite, Pt_2_HgSe_3_, a new platinum-group mineral species from the Caue iron-ore deposit, Itabira District, Minas Gerais, Brazil. *Can. Mineral.* **50**, 431–440 (2012).

[18] Marrazzo, A., Marzari, N. & Gibertini, M. Emergent dual topology in the three-dimensional Kane-Mele Pt_2_HgSe_3_. *Phys. Rev. Res.* **2**, 012–063 (2020).

[19] Cucchi, I. et al. Bulk and surface electronic structure of the dual-topology semimetal Pt_2_HgSe_3_. *Phys. Rev. Lett.* **124**, 106–402 (2020). 10.1103/PhysRevLett.124.106402 32216410

[20] de Lima, F. C., Miwa, R. H. & Fazzio, A. Jacutingaite-family: a class of topological materials. *Phys. Rev. B* **102**, 235153 (2020).

[21] Marrazzo, A., Gibertini, M., Campi, D., Mounet, N. & Marzari, N. Prediction of a large-gap and switchable Kane-Mele quantum spin hall insulator. *Phys. Rev. Lett.* **120**, 117701 (2018). 29601749 10.1103/PhysRevLett.120.117701

[22] Kandrai, K. et al. Signature of large-gap quantum spin hall state in the layered mineral jacutingaite. *Nano Lett.* **20**, 5207–5213 (2020). 32551708 10.1021/acs.nanolett.0c01499 PMC7349644

[23] Bernevig, B. A., Hughes, T. L. & Zhang, S.-C. Quantum spin Hall effect and topological phase transition in HgTe quantum wells. *Science* **314**, 1757–1761 (2006). 17170299 10.1126/science.1133734

[24] König, M. et al. Quantum spin hall insulator state in HgTe quantum wells. *Science* **318**, 766–770 (2007). 17885096 10.1126/science.1148047

[25] Roth, A. et al. Nonlocal transport in the quantum spin hall state. *Science* **325**, 294–297 (2009). 19608911 10.1126/science.1174736

[26] Knez, I., Du, R.-R. & Sullivan, G. Evidence for helical edge modes in inverted InAs/GaSb quantum wells. *Phys. Rev. Lett.* **107**, 136603 (2011). 22026882 10.1103/PhysRevLett.107.136603

[27] Wu, S. et al. Observation of the quantum spin hall effect up to 100 kelvin in a monolayer crystal. *Science* **359**, 76–79 (2018). 29302010 10.1126/science.aan6003

[28] Fei, Z. et al. Edge conduction in monolayer WTe_2_. *Nat. Phys.* **13**, 677–682 (2017).

[29] Liu, C., Culcer, D., Wang, Z., Edmonds, M. T. & Fuhrer, M. S. Helical edge transport in millimeter-scale thin films of Na_3_Bi. *Nano Lett.* **20**, 6306–6312 (2020). 32841034 10.1021/acs.nanolett.0c01649

[30] Kim, S. H. et al. Topological phase transition and quantum spin hall edge states of antimony few layers. *Sci. Rep.* **6**, 33193 (2016). 27624972 10.1038/srep33193 PMC5021940

[31] Tang, S. et al. Quantum spin hall state in monolayer 1T’-WTe_2_. *Nat. Phys.* **13**, 683–687 (2017).

[32] Reis, F. et al. Bismuthene on a SiC substrate: a candidate for a high-temperature quantum spin Hall material. *Science* **357**, 287–290 (2017). 28663438 10.1126/science.aai8142

[33] Jia, Z.-Y. et al. Direct visualization of a two-dimensional topological insulator in the single-layer 1T’-WTe_2_. *Phys. Rev. B* **96**, 041–108 (2017).

[34] Deng, J. et al. Epitaxial growth of ultraflat stanene with topological band inversion. *Nat. Mater.* **17**, 1081–1086 (2018). 30397308 10.1038/s41563-018-0203-5

[35] Shi, Y. et al. Imaging quantum spin hall edges in monolayer WTe_2_. *Sci. Adv.* **5**, eaat8799 (2019). 30783621 10.1126/sciadv.aat8799 PMC6368433

[36] Zhu, S.-Y. et al. Evidence of topological edge states in buckled antimonene monolayers. *Nano Lett.* **19**, 6323–6329 (2019). 31431010 10.1021/acs.nanolett.9b02444

[37] Stühler, R. et al. Tomonaga-Luttinger liquid in the edge channels of a quantum spin hall insulator. *Nat. Phys.* **16**, 47–51 (2020).

[38] Shumiya, N. et al. Evidence of a room-temperature quantum spin hall edge state in a higher-order topological insulator. *Nat. Mater.* **21**, 1111–1115 (2022). 35835819 10.1038/s41563-022-01304-3

[39] Maximenko, Y. et al. Nanoscale studies of electric field effects on monolayer 1T’-wte_2_. *npj Quantum Mater.* **7**, 29 (2022).

[40] Jia, J. et al. Tuning the many-body interactions in a helical Luttinger liquid. *Nat. Commun.* **13**, 6046 (2022). 36266271 10.1038/s41467-022-33676-0 PMC9584911

[41] Ghosal, C., Gruschwitz, M., Koch, J., Gemming, S. & Tegenkamp, C. Proximity-induced gap opening by twisted plumbene in epitaxial graphene. *Phys. Rev. Lett.* **129**, 116802 (2022). 36154419 10.1103/PhysRevLett.129.116802

[42] Que, Y. et al. A gate-tunable ambipolar quantum phase transition in a topological excitonic insulator. *Adv. Mater.* **36**, 2309356 (2024). 10.1002/adma.202309356 38010877

[43] Xu, Y.-J. et al. Realization of monolayer ZrTe_5_ topological insulators with wide band gaps. *Nat. Commun.* **15**, 4784 (2024). 38839772 10.1038/s41467-024-49197-x PMC11153644

[44] Leis, A. et al. Probing edge state conductance in ultra-thin topological insulator films. *Adv. Quantum Technol.* **5**, 2200043 (2022).

[45] Juergens, S., Michetti, P. & Trauzettel, B. Plasmons due to the interplay of Dirac and Schrödinger fermions. *Phys. Rev. Lett.* **112**, 076804 (2014). 24579625 10.1103/PhysRevLett.112.076804

[46] Zhang, F., Zhou, J., Xiao, D. & Yao, Y. Tunable intrinsic plasmons due to band inversion in topological materials. *Phys. Rev. Lett.* **119**, 266804 (2017). 29328685 10.1103/PhysRevLett.119.266804

[47] Duan, H.-J. et al. Bulk RKKY signatures of topological phase transition in silicene. *Sci. Rep.* **8**, 6185 (2018). 29670198 10.1038/s41598-018-24567-w PMC5906674

[48] Yu, J. & Liu, C.-X. Piezoelectricity and topological quantum phase transitions in two-dimensional spin-orbit coupled crystals with time-reversal symmetry. *Nat. Commun.* **11**, 2290 (2020). 32385246 10.1038/s41467-020-16058-2 PMC7210280

[49] Peng, R. & Liu, J. Topological piezoelectric response in moiré graphene systems. *Phys. Rev. Res.* **4**, L032006 (2022).

[50] Fachin, P., Macheda, F., Barone, P. & Mauri, F. Nearly quantized Born effective charges as probes for the topological phase transition in the Haldane and Kane-Mele models. *Phys. Rev. B* **110**, L201405 (2024).

[51] Bistoni, O., Barone, P., Cappelluti, E., Benfatto, L. & Mauri, F. Giant effective charges and piezoelectricity in gapped graphene. *2D Mater.* **6**, 045015 (2019).

[52] Binci, L., Barone, P. & Mauri, F. First-principles theory of infrared vibrational spectroscopy of metals and semimetals: application to graphite. *Phys. Rev. B* **103**, 134304 (2021).

[53] Liu, C.-C., Jiang, H. & Yao, Y. Low-energy effective Hamiltonian involving spin-orbit coupling in silicene and two-dimensional germanium and tin. *Phys. Rev. B* **84**, 195430 (2011).

[54] Nair, R. R. et al. Fine structure constant defines visual transparency of graphene. *Science* **320**, 1308–1308 (2008). 18388259 10.1126/science.1156965

[55] Kuzmenko, A. B., van Heumen, E., Carbone, F. & van der Marel, D. Universal optical conductance of graphite. *Phys. Rev. Lett.* **100**, 117–401 (2008). 10.1103/PhysRevLett.100.117401 18517825

[56] Guandalini, A. et al. Direct observation of the vanishing electron energy loss spectroscopy cross section in graphene. *Phys. Rev. B* **111**, L041401 (2025).

[57] Ezawa, M. Monolayer topological insulators: silicene, germanene, and stanene. *J. Phys. Soc. Jpn.* **84**, 121003 (2015).

[58] Haldane, F. D. M. Model for a quantum Hall effect without Landau levels: condensed-matter realization of the parity anomaly. *Phys. Rev. Lett.* **61**, 2015–2018 (1988). 10038961 10.1103/PhysRevLett.61.2015

[59] Di Sante, D., Stroppa, A., Barone, P., Whangbo, M.-H. & Picozzi, S. Emergence of ferroelectricity and spin-valley properties in two-dimensional honeycomb binary compounds. *Phys. Rev. B* **91**, 161401 (2015).

[60] Liu, C.-C., Feng, W. & Yao, Y. Quantum spin hall effect in silicene and two-dimensional germanium. *Phys. Rev. Lett.* **107**, 076802 (2021). 10.1103/PhysRevLett.107.076802 21902414

[61] Matthes, L. & Bechstedt, F. Influence of edge and field effects on topological states of germanene nanoribbons from self-consistent calculations. *Phys. Rev. B* **90**, 165431 (2014).

[62] Marzari, N., Mostofi, A. A., Yates, J. R., Souza, I. & Vanderbilt, D. Maximally localized Wannier functions: theory and applications. *Rev. Mod. Phys.* **84**, 1419–1475 (2012).

[63] Longuinhos Monteiro Lobato, R. & Ribeiro-Soares, J. Mechanical properties of layered tilkerodeite (Pd_2_HgSe_3_) and jacutingaite (Pt_2_HgSe_3_) crystals: Insights on the interlayer, intralayer interactions, and phonons. *J. Appl. Phys.* **130**, 015105 (2021).

[64] Longuinhos, R., Vymazalová, A., Cabral, A. R. & Ribeiro-Soares, J. Raman spectrum of layered tilkerodeite (Pd_2_HgSe_3_) topological insulator: the palladium analogue of jacutingaite (Pt_2_HgSe_3_). *J. Phys.: Condens. Matter* **33**, 065401 (2020). 10.1088/1361-648X/abc35a 33086198

[65] Longuinhos, R. et al. Raman spectrum of layered jacutingaite (Pt_2_HgSe_3_) crystals—experimental and theoretical study. *J. Raman Spectrosc.* **51**, 357–365 (2020).

[66] Cappelluti, E., Benfatto, L., Manzardo, M. & Kuzmenko, A. B. Charged-phonon theory and Fano effect in the optical spectroscopy of bilayer graphene. *Phys. Rev. B* **86**, 115439 (2012).

[67] Fano, U. Effects of configuration interaction on intensities and phase shifts. *Phys. Rev.* **124**, 1866–1878 (1961).

[68] Zhang, J. M. et al. Optical phonons in isotopic Ge studied by Raman scattering. *Phys. Rev. B* **57**, 1348–1351 (1998).

[69] Wirth, K. G. et al. Tunable s-SNOM for nanoscale infrared optical measurement of electronic properties of bilayer graphene. *ACS Photonics* **8**, 418–423 (2021). 33763503 10.1021/acsphotonics.0c01442 PMC7976599

[70] Wehmeier, L. et al. Landau-phonon polaritons in Dirac heterostructures. *Sci. Adv.* **10**, eadp3487 (2024). 39270026 10.1126/sciadv.adp3487 PMC11397481

[71] Ghosez, P. et al. Dynamical atomic charges: the case of ABO3 compounds. *Phys. Rev. B* **58**, 6224–6240 (1998).

[72] Villani, S. P. et al. Giant piezoelectricity driven by Thouless pump in conjugated polymers. *npj Comput. Mater.* **10**, 81 (2024).

[73] Klaassen, D. J. et al. Realization of a one-dimensional topological insulator in ultrathin germanene nanoribbons. *Nat. Comm.* **16**, 2059 (2025). 10.1038/s41467-025-57147-4 PMC11871225 40021624

[74] Giannozzi, P. et al. Advanced capabilities for materials modelling with Quantum ESPRESSO. *J. Phys. Condens. Matter* **29**, 465901 (2017). 29064822 10.1088/1361-648X/aa8f79

[75] Perdew, J. P., Burke, K. & Ernzerhof, M. Generalized gradient approximation made simple. *Phys. Rev. Lett.* **77**, 3865–3868 (1996). 10062328 10.1103/PhysRevLett.77.3865

[76] van Setten, M. et al. The PseudoDojo: training and grading a 85 element optimized norm-conserving pseudopotential table. *Comput. Phys. Commun.* **226**, 39–54 (2018).

[77] Hamann, D. R. Optimized norm-conserving Vanderbilt pseudopotentials. *Phys. Rev. B* **88**, 085117 (2013).

[78] Graziotto, L., Macheda, F., Sohier, T., Calandra, M. & Mauri, F. Theory of infrared double-resonance Raman spectrum in graphene: the role of the zone-boundary electron-phonon enhancement. *Phys. Rev. B* **109**, 075420 (2024).

[79] Monkhorst, H. J. & Pack, J. D. Special points for Brillouin-zone integrations. *Phys. Rev. B* **13**, 5188–5192 (1976).

[80] Pizzi, G. et al. Wannier90 as a community code: new features and applications. *J. Phys. Condens. Matter* **32**, 165902 (2020). 31658458 10.1088/1361-648X/ab51ff

[81] Sohier, T., Calandra, M. & Mauri, F. Density functional perturbation theory for gated two-dimensional heterostructures: theoretical developments and application to flexural phonons in graphene. *Phys. Rev. B* **96**, 075448 (2017).

[82] Venezuela, P., Lazzeri, M. & Mauri, F. Theory of double-resonant Raman spectra in graphene: intensity and line shape of defect-induced and two-phonon bands. *Phys. Rev. B* **84**, 035433 (2011).

[83] Piscanec, S., Lazzeri, M., Mauri, F., Ferrari, A. C. & Robertson, J. Kohn anomalies and electron-phonon interactions in graphene. *Phys. Rev. Lett.* **93**, 185503 (2004). 15525177 10.1103/PhysRevLett.93.185503

