# Supplementary material for: Infrared markers of topological phase transitions in quantum spin Hall insulators
Source: NPJ Comput Mater. 2025 Oct 16;11(1):307. doi: 10.1038/s41524-025-01780-6 (PMC12532721; doi:10.1038/s41524-025-01780-6)
Supplement: Supplementary file 1 — Supplementary Information [file 41524_2025_1780_MOESM1_ESM.pdf]

Supplementary Material

Paolo Fachin[1], Francesco Macheda[1], Paolo Barone[1,2], and Francesco Mauri[1,3]

[1] *Dipartimento di Fisica, Sapienza Università di Roma, Roma, Italy*
[2] *CNR-SPIN, Area della Ricerca di Tor Vergata, Roma, Italy*
[3] *Istituto Italiano di Tecnologia,Graphene Labs,Via Morego 30Genoa,I-16163Italy*

1 Kane-Mele model for buckled structures

A system described by a 2D honeycomb lattice with a buckled structure and a relevant spin-orbit coupling, such as germanene, is described by a Kane-Mele model [1] with an additional term, the intrinsic Rashba, arising from the spin-orbit coupling in non planar honeycomb lattices breaking the in-plane mirror symmetry because of the buckled structure [2], [3]. Including the effect of an external uniform electric field orthogonal to the plane of the 2D layer and assuming a zero relative on-site energy on the atoms, the complete Hamiltonian reads, according to the convention of the main text,

$$\begin{aligned} H_{\mathrm{el}} =& edE_z^{\mathrm{eff}} \sum_{i\rho} l_i c^\dagger_{i,\rho} c_{i,\rho} - t_1 \sum_{\rho} \sum_{\langle ij \rangle} c^\dagger_{i,\rho} c_{j,\rho} + i \frac{\lambda_{\mathrm{SO}}}{3\sqrt{3}} \sum_{\rho\rho'} \sum_{\langle\langle ij \rangle\rangle} l_{ij} c^\dagger_{i,\rho} (\sigma_z^{\mathrm{S}}) \rho\rho' c_{j,\rho'} \\ & - i\lambda_{\mathrm{R}} \sum_{\rho\rho'} \sum_{\langle\langle ij \rangle\rangle} l_i \, c^\dagger_{i,\rho} \left[\left(\hat{\boldsymbol{b}}_{ij} \times \hat{\boldsymbol{z}} \right) \cdot \boldsymbol{\sigma}^{\mathrm{S}} \right]_{\rho\rho'} c_{j,\rho'} \end{aligned} \tag{S1}$$

where $\hat{\boldsymbol{b}}_{ij}$ is the unit vector connecting the i and j sites, $\hat{\boldsymbol{z}}$ is the unit vector in the out plane direction and λ_{R} is the intrinsic Rashba term, introducing an off diagonal spin mixing contribution. The latter term is different from the extrinsic Rashba

$$H_{\mathrm{R}} = i\lambda'_{\mathrm{R}} \sum_{\rho\rho'} \sum_{\langle ij \rangle} l_i \, c^\dagger_{i,\rho} \left[\left(\hat{\boldsymbol{b}}_{ij} \times \hat{\boldsymbol{z}} \right) \cdot \boldsymbol{\sigma}^{\mathrm{S}} \right]_{\rho\rho'} c_{j,\rho'}, \tag{S2}$$

deriving from the presence of an external electric field or the interaction with a substrate. The only formal difference between the intrinsic and extrinsic Rashba term is that the sum is carried out either on first nearest neighbour or on next-to-nearest neighbours. The extrinsic term, which is non vanishing at the $\boldsymbol{K}$ point [1] is smaller that the intrinsic one [2] and does not affect the presence of the QSHI as well. The low energy approximation of the Kane-Mele model expanding the Hamiltonian for small quasi-momenta $\boldsymbol{p}$ around the $\boldsymbol{K}$ and $\boldsymbol{K'}$ points leads to [3]

$$H_\eta = \hbar v_{\mathrm{F}} (p_x \sigma_x^{\mathrm{P}} - \eta p_y \sigma_y^{\mathrm{P}}) + \eta \sigma_z^{\mathrm{P}} (-\lambda_{\mathrm{SO}} \sigma_z^{\mathrm{S}} - a\lambda_{\mathrm{R}} (p_y \sigma_x - p_x \sigma_y) + edE_z^{\mathrm{eff}} \sigma_z^{\mathrm{P}} \tag{S3}$$

In detail, the 4×4 Hamiltonian for $\eta = +1$ in the basis generated by $\boldsymbol{\sigma}^{\mathrm{P}} \otimes \boldsymbol{\sigma}^{\mathrm{S}}$ reads

$$H_+ = \begin{pmatrix} -\lambda_{\mathrm{SO}} + edE_z^{\mathrm{eff}} & \hbar v_{\mathrm{F}}(p_x + ip_y) & a\lambda_{\mathrm{R}}(p_y + ip_x) & 0 \\ \hbar v_{\mathrm{F}}(p_x - ip_y) & \lambda_{\mathrm{SO}} - edE_z^{\mathrm{eff}} & 0 & -a\lambda_{\mathrm{R}}(p_y + ip_x) \\ a\lambda_{\mathrm{R}}(p_y - ip_x) & 0 & \lambda_{\mathrm{SO}} + edE_z^{\mathrm{eff}} & \hbar v_{\mathrm{F}}(p_x + ip_y) \\ 0 & -a\lambda_{\mathrm{R}}(p_y - ip_x) & \hbar v_{\mathrm{F}}(p_x - ip_y) & -\lambda_{\mathrm{SO}} - edE_z^{\mathrm{eff}} \end{pmatrix} \tag{S4}$$

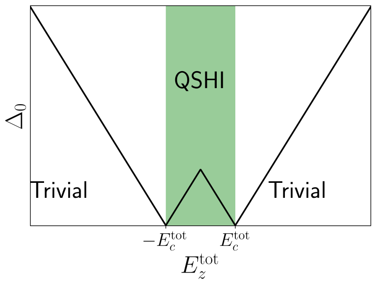

Supplementary Figure 1: Qualitative description of the topological phase transition from Quantum Spin Hall insulating phase to trivial phase driven by an orthogonal external electric field marked by the gap closure and reopening at the $\boldsymbol{K}$ and $\boldsymbol{K'}$ points

In absence of electric field the spectrum is

$$E(\boldsymbol{p}) = \pm\sqrt{\left[\hbar^2 v_{\rm F}^2 + a^2\lambda_{\rm R}^2\right] p^2 + \lambda_{\rm SO}^2} \tag{S5}$$

while with the inclusion of the electric field the energy is

$$E_\eta(\boldsymbol{p}) = \pm\sqrt{\hbar^2 v_{\rm F}^2 p^2 + \left(edE_z^{\rm eff} - \eta\sqrt{\lambda_{\rm SO}^2 + a^2\lambda_{\rm R}^2 p^2}\right)^2}. \tag{S6}$$

The intrinsic Rashba coupling has a negligible effect on the properties of the system because it vanishes at the $\boldsymbol{K}$ and $\boldsymbol{K'}$ points and it is usually much smaller than the Fermi velocity (it has a value of ~ 10 meV[2] for germanene), thus it can be neglected for the purpose of studying the topological properties of the material.

1.1 Application to germanene, stanene and silicene

The physics around the Fermi level, determining the topological phases, of germanene, as well as other similar monoelemental system from the VI group of the periodic table such as stanene and silicene composed by thin and silicon atoms, is described by the Kane-Mele model. The *ab intio* optimal parameter for the structure and the relative gap at the $\boldsymbol{K}$ point, increasing with the atomic number of the elements in the compound because of the larger spin-orbit coupling, are reported in Table 1.

	a[Å]	2d[Å]	gap [meV]
silicene	3.870	0.454	1.6
germanene	4.048	0.679	24.6
stanene	4.679	0.854	77.0

Supplementary Table 1: Lattice parameter and buckling height of free standing silicene, germanene and stanene

The parameters for the tight-binding model, described by (S1) without the intrinsic Rashba term, are obtained by fitting the *ab-initio* conduction and valence bands in absence of external field around $\boldsymbol{K}$ point along the high symmetry path $\boldsymbol{M} \to \boldsymbol{K} \to \boldsymbol{\Gamma}$ as plotted in Figure 2. The fitted values for germanene and stanene are reported in Table 2.

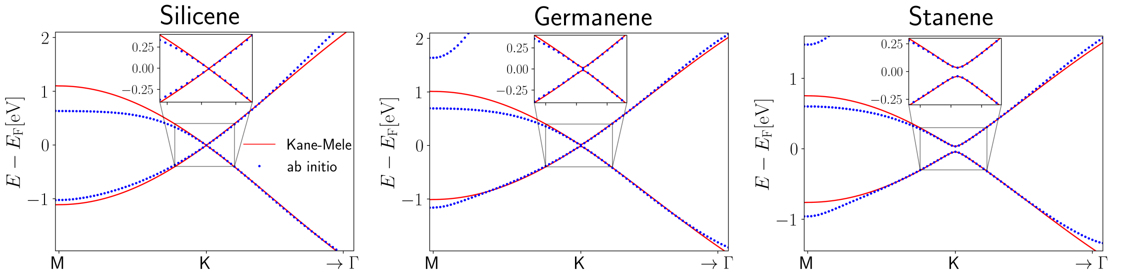

Supplementary Figure 2: *Ab initio* energy bands around the Fermi level computed, for null external electric field, compared with the Kane-Mele tight binding model with the fitted parameters indicated in Table 2 for silicene (left), germanene (centre) and stanene (right).

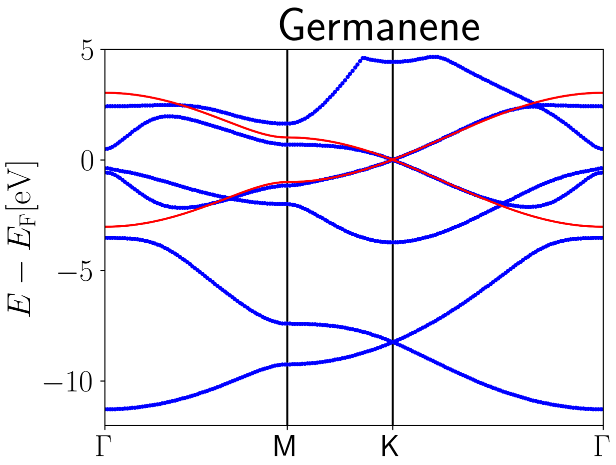

Supplementary Figure 3: *Ab initio* germanene bands and Kane-Mele description of valence and conduction bands. The Kane-Mele model provides an excellent description of the properties originating from the low energy physics.

	t_1[eV]	λ_{SO}[meV]
silicene	1.10	1.6
germanene	1.01	24
stanene	0.76	76

Supplementary Table 2: Kane-Mele tight-binding model parameters fitted from the *ab initio* bands

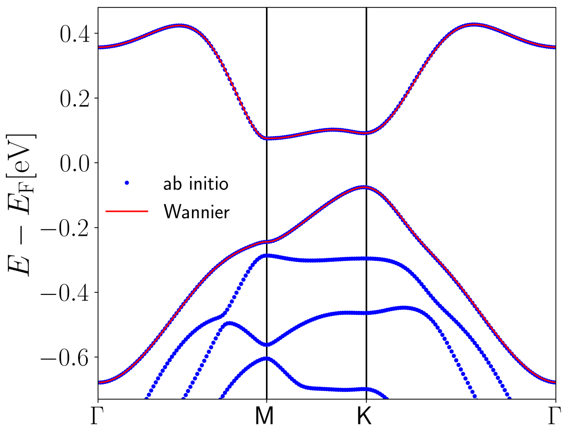

Supplementary Figure 4: The *ab initio* jacutingaite bands around the Fermi level describing the topological properties of the systems are perfectly reproduced by a 2×2 maximally localized Wannier function Hamiltonian

1.2 Application to jacutingaite

In jacutingaite, the Kane-Mele model is multiorbital, and can be derived by the use of Maximally localized Wannier functions, as discussed in the main text. We here report, in Fig. 4, the Wannierization of the band structure, obtained using 2 Wannier functions and as explained in the computational details.

2 Born Effective charges for germanene, stanene and silicene

2.1 Born Effective charges in a tight-binding approach

The Born effective charges can be decomposed into two contributions, the one due to the rigid displacement of the ionic charge Z_{ion} and the other accounting for the polarization of the electronic charge density induced by the lattice vibrations.

$$Z^*_{s,\alpha\beta} = Z^{*,\text{rig}}\delta_{\alpha\beta} + Z^{*,\text{an}}_{s,\alpha\beta}. \tag{S7}$$

Following the conventions adopted in the Supplementary of Ref.[4], the ionic contribution to the Born Effective charges, also known as Mulliken charges, reads

$$Z^{*,\text{rig}} = Z_{\text{nuc}} + \rho_s \tag{S8}$$

where Z_{nuc} is the nuclear charge, opposite to the electronic one in the tight-binding model, and ρ_s the electronic density on the s ionic site. The ionic contribution is small compared with the electronic one and has a trivial behaviour as reported in the Supplementary of Ref.[4].

2.2 Static Born Effective charges in stanene and silicene

Since stanene and silicene are described by the same model of germanene, the Born effective charges follows the same beahviour as plotted in Figure 5 where the *ab initio* simulation are compared with the numerical results of the Kane-Mele tight-binding model with the electron-phonon coupling

computed *ab initio* (Table 3). The Born effective charges are quite close in spite of the larger nearest-neighbour distance since the decrease of the electron-phonon coupling is roughly compensated by the area rescaling.

	$\xi[1/\text{Å}^2]$	Z^* trivial
silicene	0.489	2.01
germanene	0.394	1.76
stanene	0.294	1.76

Supplementary Table 3: *Ab initio* electron-phonon coupling parameter ξ and the related low energy model Born effective charges in the trivial phase

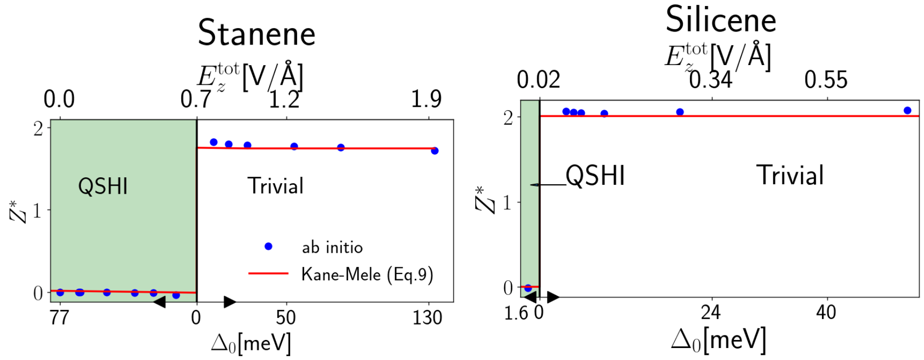

Supplementary Figure 5: Comparison between *ab initio* prediction of the Born effective charges of stanene (on the left) and silicene (on the right) and the result of Kane-Mele model with the parameters obtained from the *ab initio* simulation for a varying electric field E_z^{eff} orthogonal to the 2D sheet that closes and reopens the gap Δ_0 at the $\boldsymbol{K}$ and $\boldsymbol{K'}$ points. Black arrows indicate increase directions for Δ_0.

3 Born effective charges in jacutingaite

3.1 Tensorial properties

As discussed in the main text, following the Wyckoff positions of the atoms as presented in Tab. 4, the Born effective charges tensors have the following shape for our chosen Cartesian reference system:

$$Z^*_{\mathrm{Pt}_1} = \begin{pmatrix} Z_{xx} & 0 & 0 \\ 0 & Z_{xx} & 0 \\ 0 & 0 & Z_{zz} \end{pmatrix} \tag{S9}$$

$$Z^*_{\mathrm{Pt}_2} = \begin{pmatrix} Z^*_{xx} & 0 & 0 \\ 0 & Z^*_{yy} & Z^*_{yz} \\ 0 & Z^*_{zy} & Z^*_{zz} \end{pmatrix} \tag{S10}$$

$$Z^*_{\mathrm{Se}} = \begin{pmatrix} Z^*_{xx} & 0 & 0 \\ 0 & Z^*_{yy} & Z^*_{yz} \\ 0 & Z^*_{zy} & Z^*_{zz} \end{pmatrix} \tag{S11}$$

$$Z^*_{\mathrm{Hg}} = \begin{pmatrix} Z^*_{xx} & 0 & 0 \\ 0 & Z^*_{xx} & 0 \\ 0 & 0 & Z^*_{zz} \end{pmatrix} \tag{S12}$$

	164	$\Longrightarrow$	156
atom	Wyckoff position		Wyckoff position
Pt_1	1a	Pt_1	1a
Pt_2	3e	Pt_2	3e
Se	6i	Se_1	3d
		Se_2	3d
Hg	2d	Hg_1	1b
		Hg_2	1c

Supplementary Table 4: Wyckoff position in the unit cell of the jacutingaite monolayer in absence of the field (left) and after the inversion symmetry breaking (right)

3.2 Continuous components of Born effective charges with external field

Atoms related by inversion symmetry are constrained to have the same Born effective charges. The application of the external field and the following atomic position rearrangement removes this constraint.

For the sake of the interpretation of the spectra we can express the Born effective charges

$$\begin{aligned} Z^*_{s,\alpha\beta}(E^{\mathrm{eff}}_z) = {} & Z^*_{s,\alpha\beta}(0) + \delta Z^*_{s,\alpha\beta}\theta(|E^{\mathrm{eff}}_z| - |E^{\mathrm{eff}}_c|) \\ & + \delta Z^{*,\mathrm{topo}}_{s,\alpha\beta}(E^{\mathrm{eff}}_z)\theta(|E^{\mathrm{eff}}_c| - |E^{\mathrm{eff}}_z|) + \delta Z^{*,\mathrm{trivial}}_{s,\alpha\beta}(E^{\mathrm{eff}}_z)\theta(|E^{\mathrm{eff}}_z| - |E^{\mathrm{eff}}_c|) \end{aligned} \tag{S13}$$

where $\delta Z^{*,\mathrm{topo}}_{s,\alpha\beta}(E^{\mathrm{eff}}_z)$ and $\delta Z^{*,\mathrm{trivial}}_{s,\alpha\beta}(E^{\mathrm{eff}}_z)$ accounts for the continuos dependence on the field in the topological phase and trivial phase and are effects beyond the Kane-Male description. Those two

terms are rather small for Hg and Pt atoms and so negligible, but not on Se atoms. Nevertheless, since for all the $\pm$ partners the sum of the components of the Born Effective charges are almost conserved, they have opposite sign. $Se_{\pm}$ move in phase in all the modes except for mode VI. For all the modes but VI, it is the sum of the charges that enters in the determination of the IR intensity. Nonetheless, the sum of the effective charges on inversion symmetry related atoms does not experience large changes varying the electric field, as observed in Figure 6. For mode VI, deviations from the simple Kane-Male behaviour are nonetheless very small.

Dropping the continuos dependence on the field, the behaviour of effective charges at the transition is described by the eqauation presented in the main text, i.e.

$$Z^*_{s,\alpha\beta}(E_z^{\text{eff}}) = Z^*_{s,\alpha\beta}(0) + \delta Z^*_{s,\alpha\beta}\theta(E_z^{\text{eff}} - E_c) \tag{S14}$$

where $\delta Z^*_{s,\alpha\beta}$ is almost equal and opposite on $\pm$ partners.

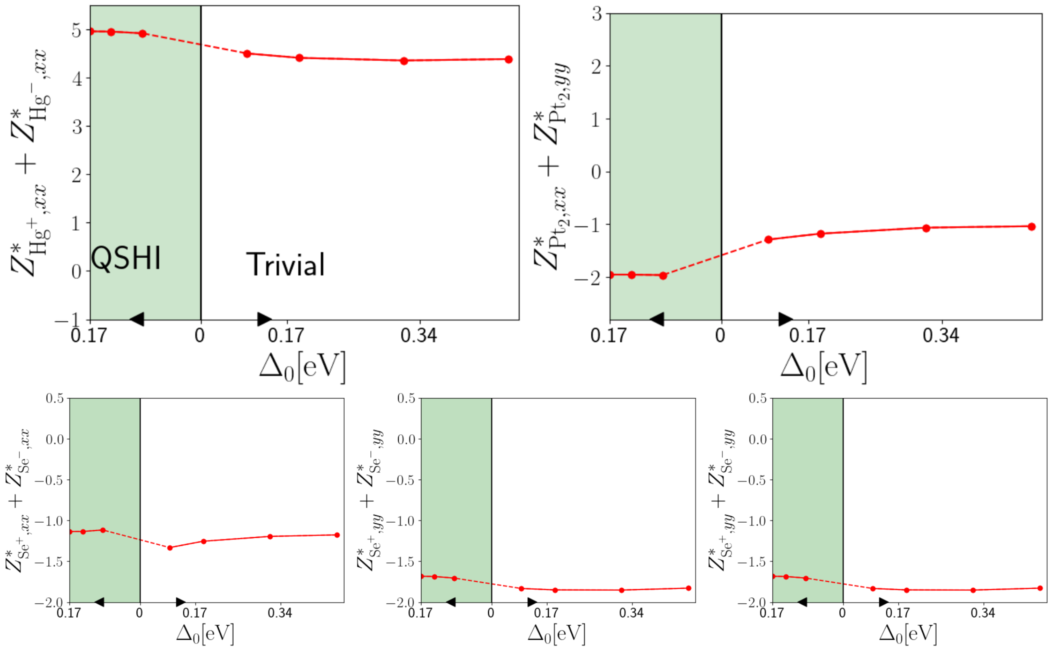

Supplementary Figure 6: Sum on inversion symmetry related atoms on jacutingaite for the components of the Born effective charges determining the infrared response as a function of the direct topological gap Δ_0, modified by the external electric field driving the system from QSHI to a trivial state. Black arrows indicate increase directions for Δ_0.

3.3 Effect of the change of the sign of the electric field

Changing the sign of the electric field, the atoms with lower and higher on-site energy exchanges and thus the contribution to the valence and conduction band wavefunction exchanges. As a consequence, the Born effective charges of the inversion symmetry related couples exchange for Hg and Se. Different Pt atoms are all on the same plane, and they are not related by inversion symmetry. So, the components of the Born effective charges do not depend on the sign of the electric field because there is no flipping of the relative on-site energy. This is coherent with what shown in Fig. 7.

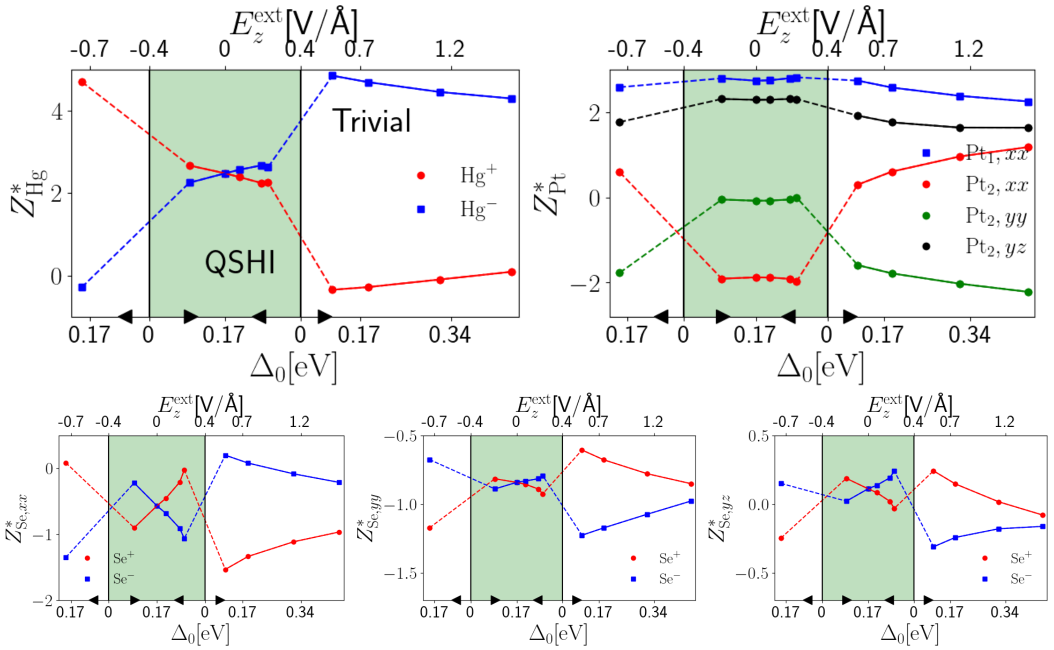

Supplementary Figure 7: Same figure as Figure 2 (c-g) in the main text, but extended to negative values of the external electric field (i.e. the one of the gate setup, not taking into account renormalization effects due to the material response). Black arrows indicate increase directions for Δ_0.

3.4 Other non zero components of the Born effective charges tensor

The out-of-plane components of the induced polarization are small with respect to the in plane ones. Those components have a trivial behaviour with respect to the topological phase transition since the out of plane response is not related to the electronic topology of the system. In addition, under exchange of the sign of the electric field, the inversion symmetry related couples exchange because of the flip in the sign of the on-site energy. By virtue of their small absolute value and their trivial beahviour across the transition, their contribution can be neglected in the study of the infrared spectra variation associated to the topological phase transition, as shown in Fig. 8.

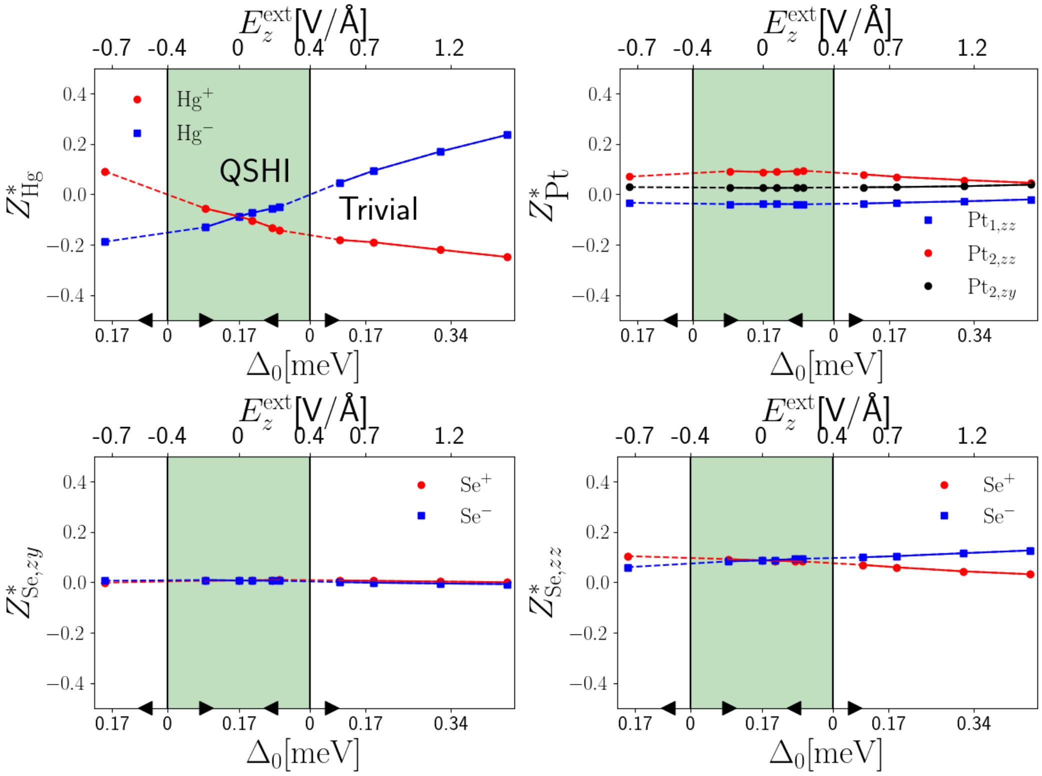

Supplementary Figure 8: Same as Fig. 7, for components of the Born effective charges that behave trivially. Black arrows indicate increase directions for Δ_0.

4 Phonon modes in jacutingaite

Jacutingaite has a 12 atom unit cell so there are 36 phonon modes, 3 acoustic, with zero frequency at zone centre, and the others are optical. In absence of an external field the system has inversion symmetry, thus there is the exclusion rule between the Raman active and the infrared active modes. Applying the external field and breaking inversion symmetry, infrared active modes becomes also Raman active and the other way round. The complete list of phonons is presented in Table 5 in the topological and trivial phases with the relative symmetry and Raman or infrared activity classification of the inversion symmetric case.

We present in Fig. 9 the evolution of the IR spectrum with different electric fields ($E_z^{\text{eff}} = 0, 1.8, 5.5, 7.4\text{V/nm}$), that highlights that changes within the same topological phase are much smaller than changes across the transition, as also shown in Tab. 6. Some of the modes with a larger infrared intensity, at least in one of the two topological phases, are plotted in Figure 10.

			Topological	- no field		Trivial	$E = 7.4$V/nm
ν			ω [meV]	IR intensity	$\rightarrow$	ω [meV]	IR intensity
1	A_{2u}	-	-0.01	0.0000		-0.01	0.0000
2-3	E_u	-	-0.01	0.0000		-0.01	0.0000
4	A_{2u}	I	3.81	0.0051		4.88	0.0042
5-6 (I)	E_u	I	6.15	0.3768		6.01	0.0310
7-8 (II)	E_g	R	7.98	0.0000		7.49	0.9564
9-10 (III)	E_u	I	8.59	1.1427		8.65	0.3775
11	A_{1g}	R	9.65	0.0000		8.96	0.0036
12	A_{2u}	I	10.42	0.0019		10.85	0.0001
13	A_{1u}	-	11.27	0.0000		11.28	0.0000
14 -15 (IV)	E_u	I	15.61	1.2454		15.35	2.6581
16-17 (V)	E_u	I	17.76	0.1973		17.73	0.0071
18	A_{2u}	I	18.08	0.0000		17.86	0.0002
19-20	E_g	R	19.53	0.0000		19.43	0.0058
21	A_{2g}	-	20.18	0.0000		20.16	0.0000
22	E_g	R	20.21	0.0000		20.19	0.0045
24	A_{1g}	R	22.50	0.0000		22.38	0.0006
25-26 (VI)	E_g	R	23.46	0.0000		23.46	0.4110
27-28 (VII)	E_u	I	24.00	0.7001		24.04	0.0110
29	A_{1g}	R	24.25	0.0000		24.23	0.0003
30	A_{1u}	-	24.53	0.0000		24.51	0.0000
31	A_{2u}	I	25.52	0.0000		25.56	0.0000
32-33(VIII)	E_u	I	25.53	0.0028		25.57	0.5171
34-35 (IX)	E_u	I	25.76	1.1746		25.79	1.2294
36	A_{2u}	I	26.64	0.0017		26.60	0.0018

Supplementary Table 5: List of *ab initio* phonon modes of jacutingaite with the symmetry classification and infrared or activity Raman activity without external field, where the system has inversion symmetry. Along with the frequency we report the infrared activity without external fields in the topological phase and in the trivial one with $E = 7.4$V/nm. IR intensities are given in units of $D^2/(\text{Å amu})$ where D stands for Debye and amu for the atomic mass unit. The Roman numerals notation connects with the one of the main text.

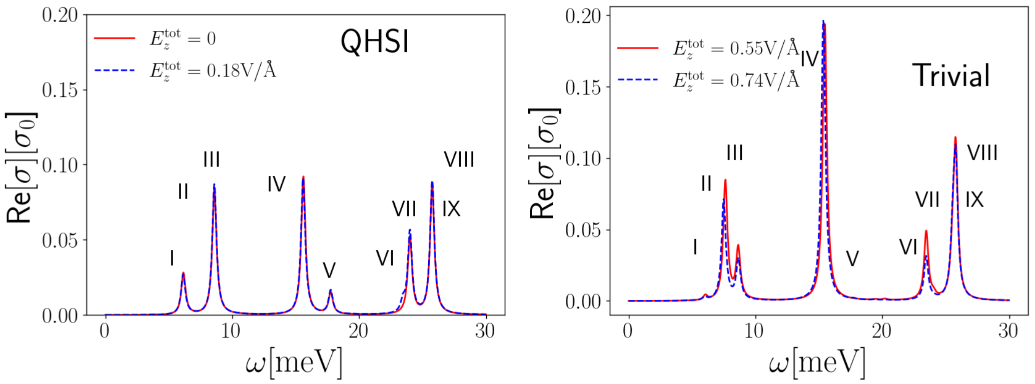

Supplementary Figure 9: On the left, the comparison between the infrared spectra taken at $E_z^{\rm eff}=0$ and $E_z^{\rm eff}=1.8$V/nm corresponding to 0.15 eV and 0.08 eV gap in the topological phase, whereas on the right the spectra are taken in the trivial phase $E_z^{\rm eff}=5.5$V/nm and $E_z^{\rm eff}=7.4$V/nm corresponding to 0.12 eV and 0.18 eV gap. The linewidth $\gamma_\nu=0.4$ meV is chosen equal for all modes for representation purposes

	topological	$E_z^{\rm eff}=0$	topological	$E_z^{\rm eff}=1.8\frac{V}{nm}$	trivial	$E_z^{\rm eff}=5.5\frac{V}{nm}$	trivial	$E_z^{\rm eff}=7.4\frac{V}{nm}$
ν	ω [meV]	IR	ω [meV]	IR	ω [meV]	IR	ω [meV]	IR
I	6.15	0.3768	6.13	0.3615	6.05	0.0457	6.01	0.0310
II	7.99	0.0000	7.94	0.0006	7.62	1.1315	7.49	0.9564
III	8.59	1.1427	8.59	1.1757	8.64	0.4833	8.65	0.3775
IV	15.61	1.2454	15.59	1.2299	15.47	2.6235	15.35	2.6581
V	17.76	0.1973	17.76	0.2133	17.75	0.0001	17.73	0.0071
VI	23.46	0.0000	23.46	0.0980	23.46	0.6491	23.46	0.4110
VII	24.01	0.7001	24.01	0.7437	24.04	0.0364	24.04	0.0110
VIII	25.53	0.0028	25.52	0.0054	25.56	0.3759	25.57	0.5171
IX	25.76	1.1746	25.77	1.1970	25.79	1.3761	25.79	1.2294

Supplementary Table 6: Comparison of the frequencies and infrared intensities of the modes with larger infrared cross section in the topological phase without external field $E_z^{\rm eff}=0$V/nm and with the external field $E_z^{\rm eff}=1.8$V/nm and in trivial phase with $E_z^{\rm eff}=5.5$V/nm and $E_z^{\rm eff}=7.4$V/nm. IR intensities are given in units of $D^2/(\text{Å amu})$ where D stands for Debye and amu for the atomic mass unit.

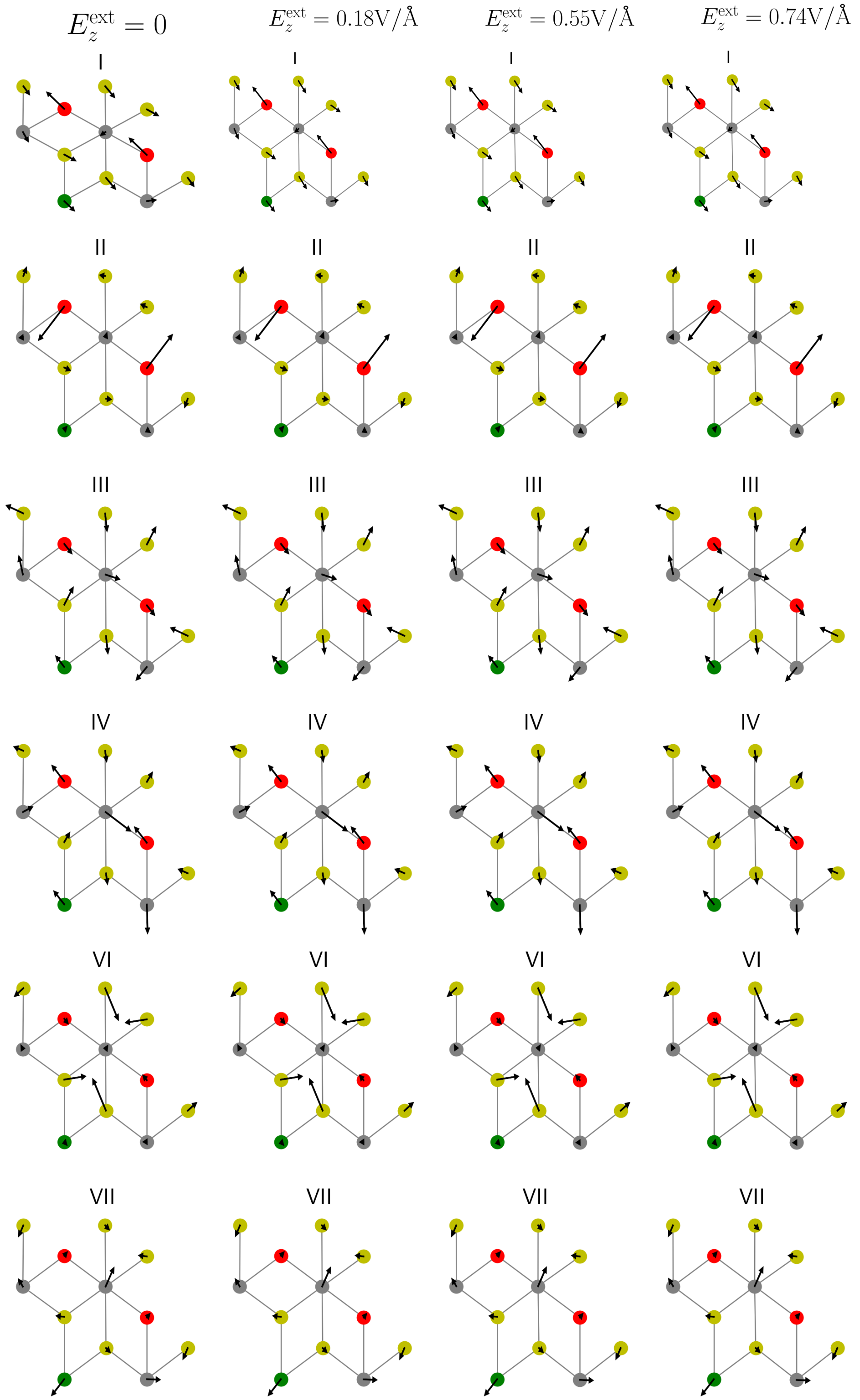

Supplementary Figure 10: Some of in-plane projected phonon modes of jacutingaite with a large infrared intensity either in the trivial or in the infrared phase for different external electric fields, $E_z^{\mathrm{eff}} = 0, 1.8\mathrm{V/nm}, 5.5\mathrm{V/nm}, 7.4\mathrm{V/nm}$ form the left to the right.

5 General equations for the optical conductivity in the low energy approximation of the Kane-Mele model

The low energy approximation describes properly system with a sufficient small gap, as for the ones considered in this work. In the approximations of this work, the Kane-Mele model reduces to two time reversal related copies of the Haldane model, one for each spin. The low energy approximation of the Haldane model allows to compute analytically the response functions even at finite frequency ω and electronic lifetime η as in the following section. For this purpose we define the following functions as in [5]

$$\begin{aligned}\tilde{\theta}(x,\omega) &= \pi - \tan^{-1}\frac{x+\hbar\omega}{\eta} - \tan^{-1}\frac{x-\hbar\omega}{\eta}\\ \tilde{\xi}(x,\omega) &= \frac{1}{2}\log\frac{(x+\hbar\omega)^2+\zeta^2}{(x-\hbar\omega)^2+\zeta^2}\end{aligned} \tag{S15}$$

and the gap at the point $\boldsymbol{K}$ and $\boldsymbol{K'}$ of each Haldane model is

$$\Delta(\eta) = edE_z^{\text{eff}} - \eta\lambda_{\text{SO}} \tag{S16}$$

where $\eta = \pm 1$ depending on the valley.

5.1 Frequency dependent Born effective charges

The frequency dependent Born effective Charges are [5]

$$Z^*_{s,\alpha\beta}(\omega) = \frac{2ie}{N_{\boldsymbol{k}}}\sum_{\boldsymbol{k},ij}\frac{f_{\boldsymbol{k},i}-f_{\boldsymbol{k},j}}{\epsilon_{\boldsymbol{k},i}-\epsilon_{\boldsymbol{k},j}+\hbar\omega+i\zeta}\langle u_{\boldsymbol{k},i}|\frac{\hbar v_\alpha}{\epsilon_{\boldsymbol{k},i}-\epsilon_{\boldsymbol{k},j}}|u_{\boldsymbol{k},j}\rangle\langle u_{\boldsymbol{k},j}|\frac{\partial H}{\partial u_{\beta,s}}|u_{\boldsymbol{k},i}\rangle \tag{S17}$$

that in the low energy approximation, thanks to the substitutions

$$\begin{aligned}v_\alpha\Big|_{\boldsymbol{K}} &\to v_{\text{F}}\sigma^{\text{P}}_\alpha\\ \frac{\partial H}{\partial u_{s,\beta}}\Big|_{\boldsymbol{K}} &\to \hbar v_{\text{F}}\frac{\beta}{b_0^2}(\sigma^{\text{P}}\times\hat{z})_\beta\\ v_\alpha\Big|_{\boldsymbol{K'}} &\to -v_{\text{F}}\left(\sigma^{\text{P}}\right)^*_\alpha\\ \frac{\partial H}{\partial u_{s,\beta}}\Big|_{\boldsymbol{K'}} &\to \hbar v_{\text{F}}\frac{\beta}{b_0^2}(\sigma^{\text{P}}\times\hat{z})^*_\beta\end{aligned} \tag{S18}$$

where b_0 nearest neighbour distance, becomes

$$Z^*_{s,\alpha\alpha} = A\sum_\eta\xi\int_{|\Delta(\eta)|/2}^{+\infty}\frac{dE}{2\pi}\frac{\Delta(\eta)}{4E}\left(\frac{1}{2E-\hbar\omega-i\zeta}+\frac{1}{2E+\hbar\omega+i\zeta}\right) \tag{S19}$$

leading to the following results

$$\begin{aligned}\text{Re}\big(Z^*_{s,\alpha\alpha}(\omega)\big) &= \sum_\eta\frac{A\xi}{2\pi}\frac{\Delta(\eta)\hbar\omega}{(\hbar\omega)^2+\zeta^2}\left[\frac{\zeta}{\hbar\omega}\tilde{\theta}(|\Delta(\eta)|,\omega)+\tilde{\xi}(|\Delta(\eta)|,\omega)\right]\\ \text{Im}\big(Z^*_{s,\alpha\alpha}(\omega)\big) &= \sum_\eta\frac{A\xi}{2\pi}\frac{\Delta(\eta)\hbar\omega}{(\hbar\omega)^2+\zeta^2}\left[\tilde{\theta}(|\Delta(\eta)|,\omega)-\frac{\eta}{\hbar\omega}\tilde{\xi}(|\Delta(\eta)|,\omega)\right]\end{aligned} \tag{S20}$$

5.2 Electronic optical conductivity

The diagonal components of the electronic optical conductivity read

$$\sigma_{\alpha\alpha}(\omega) = \sigma_0 \frac{i}{N_{\boldsymbol{k}} A} \sum_{\boldsymbol{k},ij} \frac{f_{\boldsymbol{k},i} - f_{\boldsymbol{k},j}}{\epsilon_{\boldsymbol{k},i} - \epsilon_{\boldsymbol{k},j} + \hbar\omega + i\zeta} \langle u_{\boldsymbol{k},i}| \frac{\hbar v_\alpha}{\epsilon_{\boldsymbol{k},i} - \epsilon_{\boldsymbol{k},j}} |u_{\boldsymbol{k},j}\rangle \langle u_{\boldsymbol{k},j}| \hbar v_\alpha |u_{\boldsymbol{k},i}\rangle \tag{S21}$$

that in the low energy approximation, exploiting the substitutions as in Eq. (S18)

$$\sigma_{\alpha\alpha} = i\sigma_0 \sum_\eta \int_{|\Delta(\eta)|/2}^{+\infty} \frac{dE}{2\pi} \left(1 + \frac{[\Delta(\eta)]^2}{4E^2}\right) \left(\frac{1}{2E + \hbar\omega + i\zeta} - \frac{1}{2E - \hbar\omega - i\zeta}\right) \tag{S22}$$

where $\sigma_0 = \frac{e^2}{4\hbar}$ is the universal conductivity of clean graphene. For a finite ζ the electronic contribution to the optical conductivity reads

$$\begin{aligned}
\Re(\sigma_{\alpha\alpha}) =& \frac{\sigma_0}{2\pi} \sum_\eta \Big[\tilde{\theta}(|\Delta(\eta)|, \omega) + \frac{2\zeta\omega}{\omega^2 + \zeta^2} + \\
&+ \frac{\Delta^2}{\omega^2 + \zeta^2} \Big((\omega^2 - \zeta^2)\tilde{\theta}(|\Delta(\eta)|, \omega) - 2\omega\zeta\tilde{\xi}(|\Delta(\eta)|, \omega)\Big)\Big] \\
\Im(\sigma_{\alpha\alpha}) =& \frac{\sigma_0}{2\pi} \sum_\eta \Big[- \tilde{\xi}(|\Delta(\eta)|, \omega) \left(1 + \Delta^2 \frac{\omega^2 - \zeta^2}{(\omega^2 + \zeta^2)^2}\right) - \\
&+ \frac{2\Delta^2\zeta\omega}{(\omega^2 + \zeta^2)^2} \tilde{\theta}(|\Delta(\eta)|, \omega) + 2\Delta \frac{\omega}{\omega^2 + \zeta^2}\Big]
\end{aligned} \tag{S23}$$

with the clean limit

$$\begin{aligned}
\mathrm{Re}(\sigma^{\mathrm{el}}_{xx}(\omega)) &= \sum_\eta \frac{\sigma_0}{2} \left(1 + \frac{[\Delta(\eta)]^2}{\omega^2}\right) \theta(\omega - \Delta(\eta)) \\
\mathrm{Im}(\sigma^{\mathrm{el}}_{xx}(\omega)) &= \sum_\eta \frac{\sigma_0}{2\pi} \Big[\log \frac{|\Delta(\eta)| - \omega}{|\Delta(\eta)| + \omega} \left(1 + \frac{[\Delta(\eta)]^2}{\omega^2}\right) + 2\frac{\Delta(\eta)}{\omega}\Big]
\end{aligned} \tag{S24}$$

presented in the main text. The low frequency limit of the imaginary part is

$$\begin{aligned}
\mathrm{Im}(\sigma^{\mathrm{el}}_{xx}(\omega)) &= \sum_\eta \frac{\sigma_0}{2\pi} \Big[- \left(\frac{2\omega}{|\Delta(\eta)|} + \frac{2\omega^3}{3|\Delta(\eta)|^3}\right) \left(1 + \frac{[\Delta(\eta)]^2}{\omega^2}\right) + 2\frac{\Delta(\eta)}{\omega}\Big] \\
&= -\sum_\eta \frac{\sigma_0}{\pi} \frac{4}{3} \frac{\omega}{|\Delta(\eta)|}
\end{aligned} \tag{S25}$$

Since the off resonant linear contribution in ω of the optical conductivity is related to the eletric susceptibility by

$$\mathrm{Im}(\sigma^{\mathrm{el}}(\omega)) = -i\omega\epsilon_0\chi(\omega = 0) \tag{S26}$$

it follows that

$$\chi(\omega = 0) = \frac{4}{3}\frac{1}{\epsilon_0} \sum_\eta \frac{\sigma_0}{\pi} \frac{1}{|\Delta(\eta)|} \tag{S27}$$

5.3 Phonon self-energy

The phonon-self energy reads

$$\Pi_{s\alpha,s\alpha'}(\omega) = 2\sum_{\boldsymbol{k},\boldsymbol{k'},ij}\left(\frac{f_{\boldsymbol{k'},i}-f_{\boldsymbol{k},j}}{\epsilon_{\boldsymbol{k'},i}-\epsilon_{\boldsymbol{k},j}+\omega+i\zeta}-\frac{f_{\boldsymbol{k'},i}-f_{\boldsymbol{k},j}}{\epsilon_{\boldsymbol{k'},i}-\epsilon_{\boldsymbol{k},j}}\right)\langle\psi_{\boldsymbol{k},j}|\,\frac{\partial H}{\partial u_{s,\alpha}}\,|\psi_{\boldsymbol{k'},i}\rangle\,\langle\psi_{\boldsymbol{k'},i}|\,\frac{\partial H}{\partial u_{s',\beta}}\,|\psi_{\boldsymbol{k},j}\rangle \tag{S28}$$

In an honeycomb lattice with two sublattices, A, B, describing a time reversal symmetric system where the electron-phonon coupling depends on the relative displacement of the two atoms $\boldsymbol{u}_A-\boldsymbol{u}_B$, the phonon self energy at zone centre for has only one independent value

$$\begin{aligned}\Pi_{Ax,Ax} &= \Pi_{Bx,Bx} = \Pi_{Ay,Ay} = \Pi_{By,By}\\ -\Pi_{Ax,Ax} &= \Pi_{Ax,Bx} = \Pi_{Bx,Ax} = \Pi_{By,Ay} = \Pi_{Ay,By}\\ \Pi_{Ax,Ay} &= \Pi_{Ay,Ax} = \Pi_{Bx,By} = \Pi_{By,Bx} = 0\\ \Pi_{Ax,By} &= \Pi_{By,Ax} = \Pi_{Bx,Ay} = \Pi_{Ay,Bx} = 0\end{aligned} \tag{S29}$$

In the next paragraphs, we present the analytical calculation, while the numerical results are shown in Fig. 11

5.3.1 Frequency renormalization

The hermitian part of the self energy affects the phonon frequencies as

$$\sum_{s'\alpha'}\left(C_{s\alpha,s'\alpha'}+\Pi_{s\alpha,s'\alpha'}(\omega)\right)e_{s'\alpha'} = \omega^2 e_{s\alpha} \tag{S30}$$

By virtue of the symmetries of the system the dynamical matrix and the self-energy admits the same basis of eigenvectors, the frequency is

$$\omega^2 = \omega_{\text{ph}}^2 + (\delta\omega_{\text{ph}})^2, \quad \delta\omega_{\text{ph}} = \frac{\text{Re}[\Pi_{sx,sx}(\omega_{\text{ph}})]}{\omega_{\text{ph}}} \tag{S31}$$

The real part is then

$$\text{Re}(\Pi_{xx}(\omega)) = \sum_\eta \eta\frac{A\beta^2_{\text{e-ph}}}{b_0^4}\int_{|\Delta(\eta)|/2}^{+\infty}\frac{dE}{2\pi}\left(\frac{(2E-\omega)}{(2E-\omega)^2+\zeta^2}+\frac{2E+\omega}{(2E+\omega)^2+\zeta^2}-\frac{1}{E}\right)\left(E+\frac{[\Delta(\eta)]^2}{4E}\right) \tag{S32}$$

and after integration

$$\text{Re}(\Pi_{xx}(\omega)) = \sum_\eta\frac{1}{2\pi}\frac{9\sqrt{3}\beta^2_{\text{e-ph}}}{4a^2}\Big[\left(1-\frac{[\Delta(\eta)]^2}{\omega^2+\zeta^2}\right)\eta\tilde{\theta}(|\Delta(\eta)|,\omega)+2|\Delta(\eta)|-\left(\frac{[\Delta(\eta)]^2\omega}{(\omega^2+\zeta^2)}+\omega\right)\tilde{\xi}(|\Delta(\eta)|,\omega)\Big] \tag{S33}$$

In the limit for $\zeta\to 0$ the self energy reads

$$\text{Re}(\Pi_{xx}(\omega)) = \sum_\eta\frac{1}{2\pi}\frac{9\sqrt{3}\beta^2_{\text{e-ph}}}{4a^2}\Big[2|\Delta(\eta)|-\left(\frac{[\Delta(\eta)]^2}{\omega}+\omega\right)\log\frac{(|\Delta(\eta)|+\omega)}{(|\Delta(\eta)|-\omega)}\Big] \tag{S34}$$

that presents a logarithmic divergence for $\Delta=\omega$.

5.3.2 Lifetime

The imaginary part of the phonon self-energy

$$\mathrm{Im}(\Pi_{xx}(\omega)) = \sum_\eta \eta \frac{A\beta^2_{\text{e-ph}}}{b_0^4} \int_{|\Delta(\eta)|/2}^{+\infty} \frac{dE}{2\pi} \left(\frac{1}{(2E-\omega)^2+\zeta^2} - \frac{1}{(2E+\omega)^2+\zeta^2} \right) \left(E + \frac{[\Delta(\eta)]^2}{4E} \right) \tag{S35}$$

is related to the lifetime by

$$\gamma^{\text{e-ph}} = -\frac{2\mathrm{Im}[\Pi_{xx}(\omega_{\text{ph}})]}{\hbar\omega_{\text{ph}}} \tag{S36}$$

leading to

$$\gamma^{\text{e-ph}} = \sum_\eta \eta \frac{2A}{M\omega_{\text{ph}}} \left(\frac{\beta_{\text{e-ph}}}{b_0^2} \right)^2 \int_{|\Delta(\eta)|/2}^{+\infty} \frac{dE}{2\pi} \left(\frac{1}{(2E-\omega)^2+\zeta^2} - \frac{1}{(2E+\omega)^2+\zeta^2} \right) \left(E + \frac{[\Delta(\eta)]^2}{4E} \right) \tag{S37}$$

that, integrated, is

$$\gamma^{\text{e-ph}} = \sum_\eta \bar{\gamma} \frac{1}{2\pi} \Big[\tilde{\theta}(|\Delta(\eta)|, \omega_{\text{ph}}) + \frac{\zeta}{\omega_0} \tilde{\xi}(|\Delta(\eta)|, \omega_{\text{ph}}) + \frac{|\Delta(\eta)|^2}{\omega_{\text{ph}}^2+\zeta^2} \left(\tilde{\theta}(|\Delta(\eta)|, \omega_{\text{ph}}) - \frac{\zeta}{\omega_0} \tilde{\xi}(|\Delta(\eta)|, \omega_{\text{ph}}) \right) \Big] \tag{S38}$$

where

$$\bar{\gamma} = \frac{9\sqrt{3}\beta^2_{\text{e-ph}}}{4Ma^2} \tag{S39}$$

In the $\zeta \to 0$ limit, the phonon lifetime reads

$$\gamma^{\text{e-ph}} = \bar{\gamma} \sum_\eta \left(1 + \frac{[\Delta(\eta)]^2}{(\hbar\omega_{\text{ph}})^2} \right) \theta(\hbar\omega - |\Delta(\eta)|). \tag{S40}$$

5.4 Electronic excitation effects in germanene on the electric susceptibility beyond the Kane-Mele model

The optical conductivity is related to the susceptibility in SI units by

$$\sigma(\omega) = -i\omega\epsilon_0\chi(\omega) \tag{S41}$$

The real part electronic contribution to the optical conductivity is different from zero only for frequencies excitating electronic states whereas the imaginary part is linear in ω in the off resonant regime since $\chi(\omega = 0)$ is finite. Thus, in a proper description of the dynamical effects in germanene, for the imaginary part, the contribution of the bands beyond valence and conduction, not included in the tight-binding Kane-Mele model, is included exploiting the *ab initio* simulations. For a 2D system we can derive the static real susceptibility in SI units as

$$\chi_0^{ab\ initio} = c(\epsilon - 1) \tag{S42}$$

where ϵ is the 3D static dielectric response computed in Quantum Espresso and c is the interlayer distance. Thus, we compute the optical conductivity as

$$\sigma(\omega) = -i\omega\epsilon_0(\chi^{\text{KM}}(\omega) - \chi^{\text{KM}}(0) + \chi^{ab\ initio}(0)) \tag{S43}$$

where the label KM indicates the quantities computed using the low energy Kane-Mele model according to eqs. (S24)and (S27), while the *ab initio* susceptibility is obtained using eq. (S42).

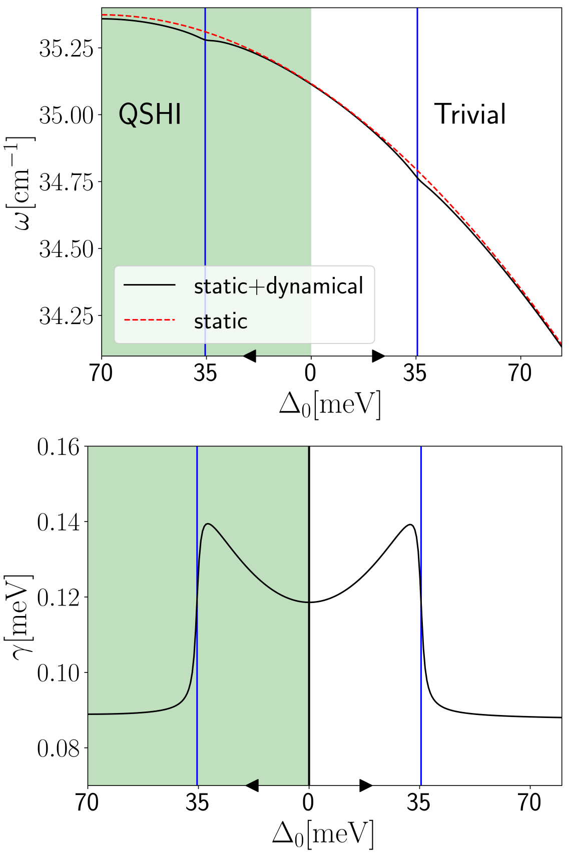

Supplementary Figure 11: Top: variation of phonon frequency by inclusion of static effects coming from the *ab-initio* variation of the adiabatic phonon frequency as a function of the gap, plus dynamical effects due to self-energy renormalization. Bottom: linewidth of the phonons as a function of the gap. Black arrows indicate increase directions for Δ_0.

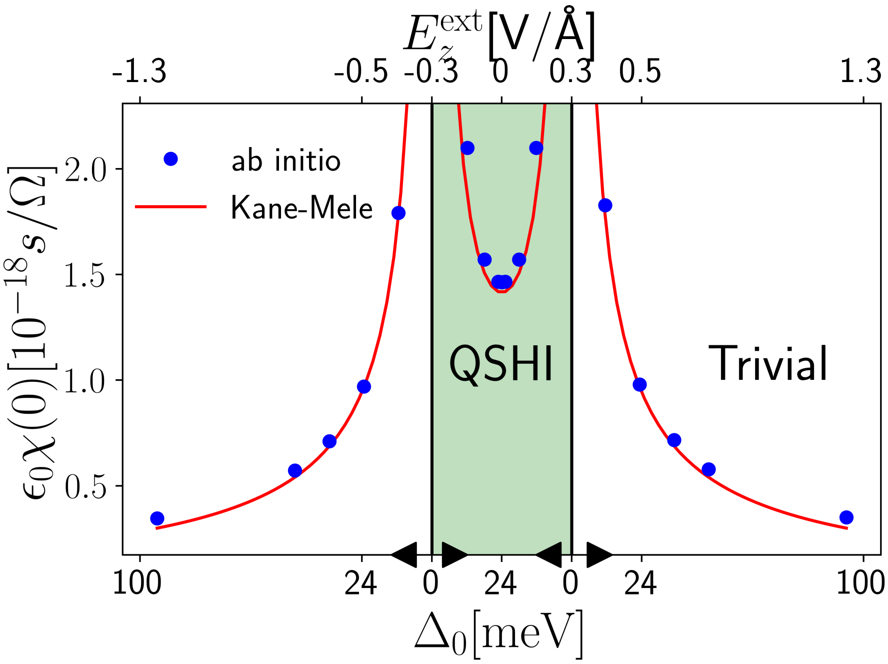

Supplementary Figure 12: Comparison between the electric susceptibility computed *ab initio* for germanene and using the low energy Kane-Mele model varying the external electric field and so the direct gap Δ_0. Black arrows indicate increase directions for Δ_0. The negligible difference confirms that the low energy physics properly describes the properties of germanene.

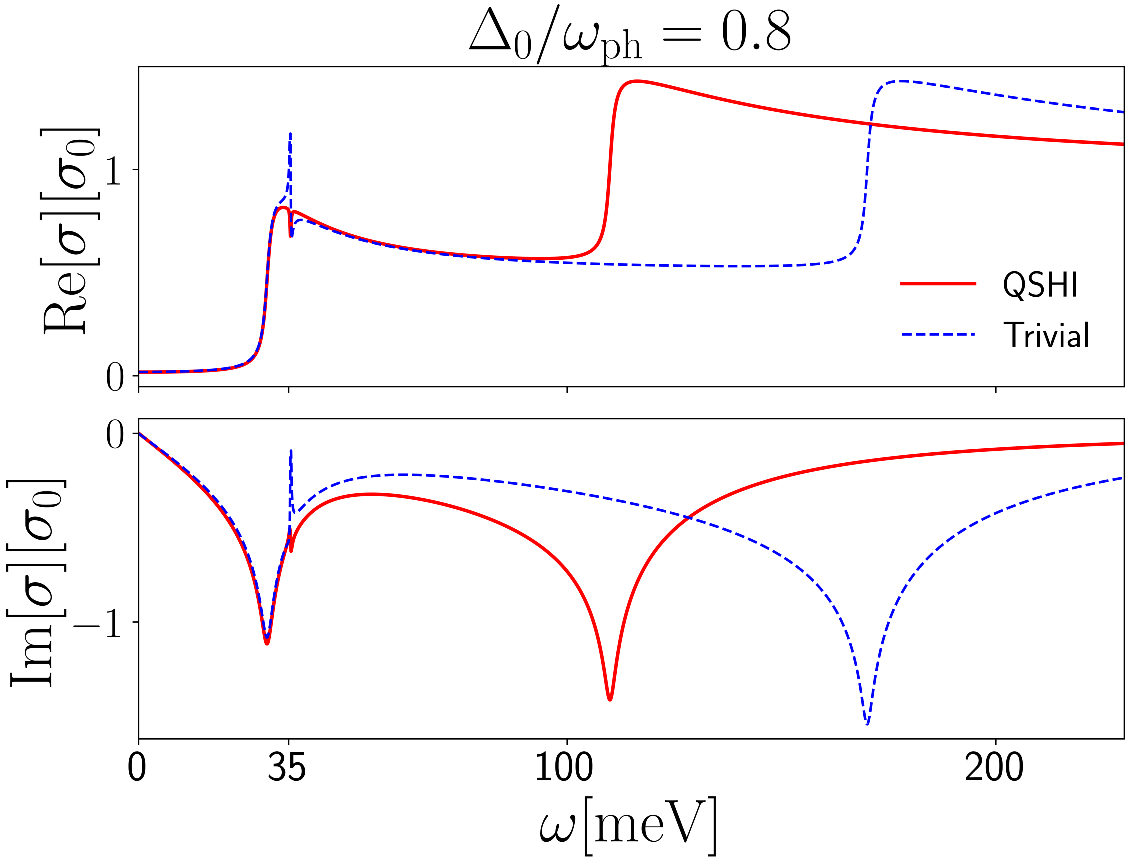

Supplementary Figure 13: Real and imaginary part of the optical conductivity showing both the phonon and the electronic contributions for the ratio between $\Delta_0/\omega_{ph} = 0.8$ in the QSHI and trivial phases. Each spectrum has two electronic excitation resonances due to the band splitting caused by the electric field, the one between valence and conduction band $\Delta_0 = 2edE_z^{\rm eff} - 2\lambda_{SO}$ and the other between the lower and the higher bands $\Delta_1 = 2edE_z^{\rm eff} + 2\lambda_{SO}$. Since the spectra are taken for the same Δ_0 in the two phases, Δ_1 differs being larger in the trivial phase.

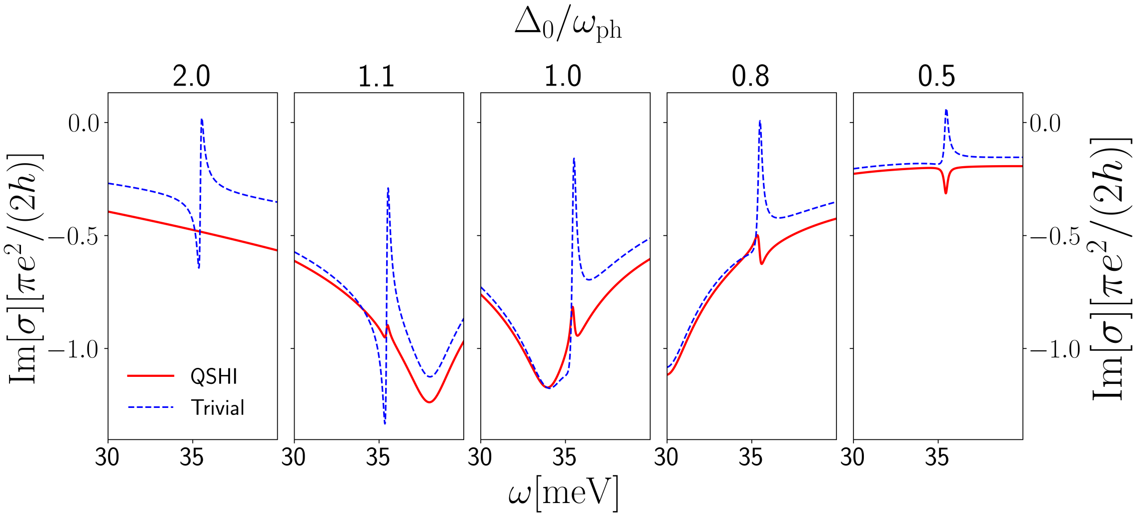

Supplementary Figure 14: Imaginary part of the optical conductivity of the Kane-Mele model, taking into account dynamical effects on both the electronic and ionic optical conductivities, for both the QSHI and the trivial phases, as a function of the ratio $\Delta_0/\omega_{\rm ph}$. Where the topological gap is much larger than the phonon frequencies, we recover the same results as for the static treatment. When the topological gap is comparable or smaller than the phonon frequencies, dynamical effects kick in, and the phonon is visible even in the QSHI phase. Nonetheless, the two phases are still very clearly distinguishable due to the different shape of the profiles.

6 Reflectivity and Transmittance spectra of germanene

The local $3m$ site-symmetries of germanene constrain the optical conductivity tensor to be diagonal with equal in-plane components ($\sigma_{xx}(\omega) = \sigma_{yy}(\omega)$). The in-plane component is referred to as $\sigma(\omega)$ in the following equations. Imposing the boundary conditions of the electromagnetic fields, the reflectivity and the transmittance of the 2D material for light polarized along one of its principal axes are respectively [6]

$$R = \left| \frac{Z_0 \sigma(\omega)}{2 + Z_0 \sigma(\omega)} \right|^2 \tag{44}$$

and

$$T = \left| \frac{2}{2 + Z_0 \sigma(\omega)} \right|^2 \tag{45}$$

where $Z_0 = \sqrt{\frac{\mu_0}{\epsilon_0}} = 377\,\Omega$. The reflectivity and transmittance spectra of germanene for different ratios between the electronic gap and the phonon frequency are reported in Fig. 15. The electronic

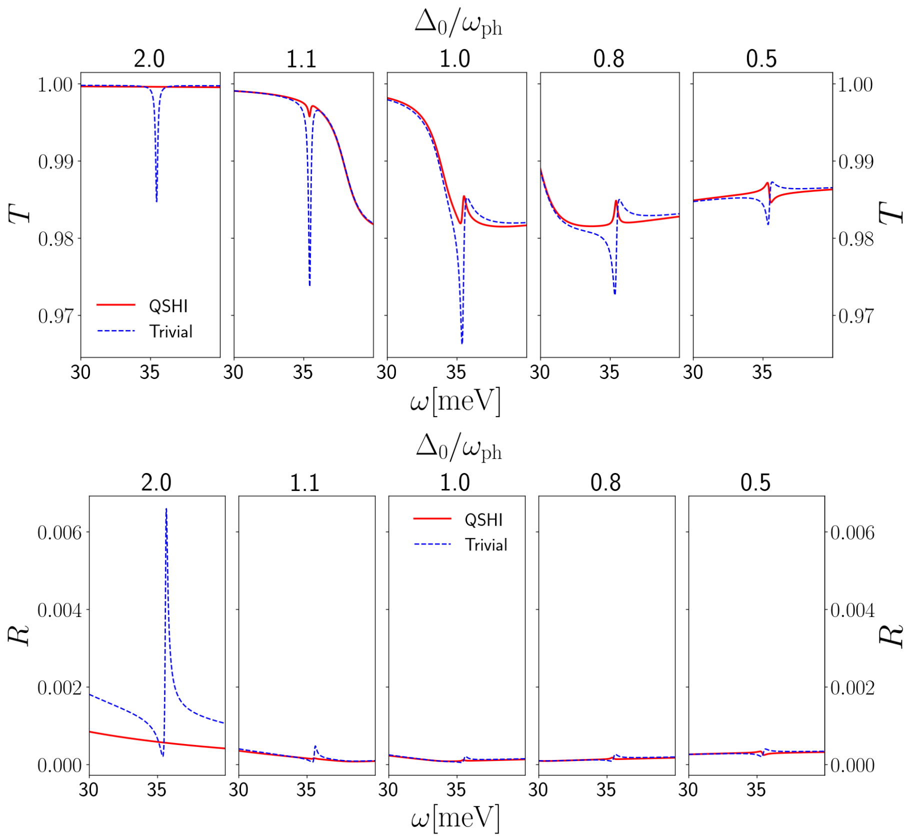

Supplementary Figure 15: Transmittance and reflectivity of a germanene sample including dynamical effects on both the electronic and ionic optical conductivities, for both the QSHI and the trivial phases, as a function of the ratio $\Delta_0/\omega_{\rm ph}$ using the Kane-Mele low energy model.

contribution to these spectra have the same order of magnitude of the one of graphene [7], [8].

References

[1] C. L. Kane and E. J. Mele, "Quantum spin hall effect in graphene," *Phys. Rev. Lett.*, vol. 95, p. 226 801, 22 2005. DOI: 10.1103/PhysRevLett.95.226801.

[2] C.-C. Liu, H. Jiang, and Y. Yao, "Low-energy effective hamiltonian involving spin-orbit coupling in silicene and two-dimensional germanium and tin," *Phys. Rev. B*, vol. 84, p. 195 430, 19 2011. DOI: 10.1103/PhysRevB.84.195430.

[3] M. Ezawa, "A topological insulator and helical zero mode in silicene under an inhomogeneous electric field," *New Journal of Physics*, vol. 14, no. 3, p. 033 003, 2012. DOI: 10.1088/1367-2630/14/3/033003.

[4] P. Fachin, F. Macheda, P. Barone, and F. Mauri, "Nearly quantized born effective charges as probes for the topological phase transition in the haldane and kane-mele models," *Phys. Rev. B*, vol. 110, p. L201405, 20 2024. DOI: 10.1103/PhysRevB.110.L201405.

[5] O. Bistoni, P. Barone, E. Cappelluti, L. Benfatto, and F. Mauri, "Giant effective charges and piezoelectricity in gapped graphene," *2D Materials*, vol. 6, no. 4, p. 045 015, 2019. DOI: 10.1088/2053-1583/ab2ce0.

[6] A. Guandalini, R. Senga, Y.-C. Lin, *et al.*, "Direct observation of the vanishing electron energy loss spectroscopy cross section in graphene," *Phys. Rev. B*, vol. 111, p. L041401, 4 2025. DOI: 10.1103/PhysRevB.111.L041401.

[7] R. R. Nair, P. Blake, A. N. Grigorenko, *et al.*, "Fine structure constant defines visual transparency of graphene," *Science*, vol. 320, no. 5881, pp. 1308–1308, 2008. DOI: 10.1126/science.1156965. eprint: https://www.science.org/doi/pdf/10.1126/science.1156965.

[8] A. B. Kuzmenko, E. van Heumen, F. Carbone, and D. van der Marel, "Universal optical conductance of graphite," *Phys. Rev. Lett.*, vol. 100, p. 117 401, 11 2008. DOI: 10.1103/PhysRevLett.100.117401.
